# Multi-region proteomic mapping identifies FTL1 and SERPINA3K as protective factors in cardiac aging

**DOI:** 10.1038/s41419-026-08882-z

**Published:** 2026-05-23

**Authors:** Jingnan Huang, Xin Sun, Huadong Liu, Kunpeng Li, Xin Liu, Yunmeng Bai, Zhiyu Dong, Xinlei Wu, Xinyi Liu, Lin Jia, Jianlong Yan, Lixin Cheng, Jigang Wang, Lingyun Dai, Qingshan Geng

**Affiliations:** 1 Guangdong Provincial Clinical Research Center for Geriatrics, Shenzhen Clinical Research Center for Geriatrics, Department of Geriatrics, Shenzhen People’s Hospital (The First Affiliated Hospital, Southern University of Science and Technology; The Second Clinical Medical College, Jinan University), Shenzhen, 518020 China; 2 Department of Cardiology, Shenzhen Cardiovascular Minimally Invasive Medical Engineering Technology Research and Development Center, Shenzhen People’s Hospital (The First Affiliated Hospital, Southern University of Science and Technology; The Second Clinical Medical College, Jinan University), Shenzhen, 518020 China; 3 Department of Pharmacology, School of Medicine and SUSTech Homeostatic Medicine Institute (SHMI), Southern University of Science and Technology, Shenzhen, 518055 China; 4 School of Software, Beihang University, Beijing, 100191 China; 5 College of Pharmacy, Shenzhen Technology University, Shenzhen, 518118 China; 6 Institute of Molecular and Cell Biology, Agency for Science, Technology and Research (A*STAR), Singapore, 138673 Singapore

**Keywords:** Proteomics, Senescence

## Abstract

Aging is a well-recognized risk factor in cardiovascular diseases (CVDs), primarily due to its association with the gradual decline in cardiac function. This decline significantly influences the pathogenesis of common CVDs such as myocardial infarction and heart failure. Despite the existence of several proteomic atlases of the heart, the spatially resolved proteomic dynamics essential for understanding region-specific aging mechanisms in cardiac tissue remain incompletely characterized. In this study, we conducted a region-resolved quantitative proteomic profiling for various murine cardiac regions at three distinct stages of aging (3, 12, and 20-month-old), quantifying 6 650 proteins in the heart. Leveraging integrated bioinformatics and machine learning frameworks, we uncovered that FTL1 and SERPINA3K exhibit strong age-associated expression changes across all cardiac regions. Mechanistically, the knockdown of *Ftl1* led to cardiomyocyte ferroptosis and senescence, phenotypes that were ameliorated by the ferroptosis inhibitor Ferrostatin-1. Furthermore, the depletion of *Serpina3k* exacerbated senescence and collagen deposition through the activation of the cGAS-STING-PERK axis, effects that can be reversed via the overexpression of *Serpina3k* or the knockdown of *Sting*. The protective effect of SERPINA3K was also demonstrated in vivo through AAV9-mediated cardiomyocyte-specific overexpression in middle-aged mice, which attenuated the cGAS-STING-PERK axis and mitigated age-related fibrosis. These results strongly demonstrated that FTL1 and SERPINA3K function as key regulators of cardiac aging. Collectively, this study provides a valuable region-resolved proteomic atlas of cardiac aging and identifies key protein regulators, thereby uncovering potential targets for cardio-protective interventions against age-related cardiovascular disorders.

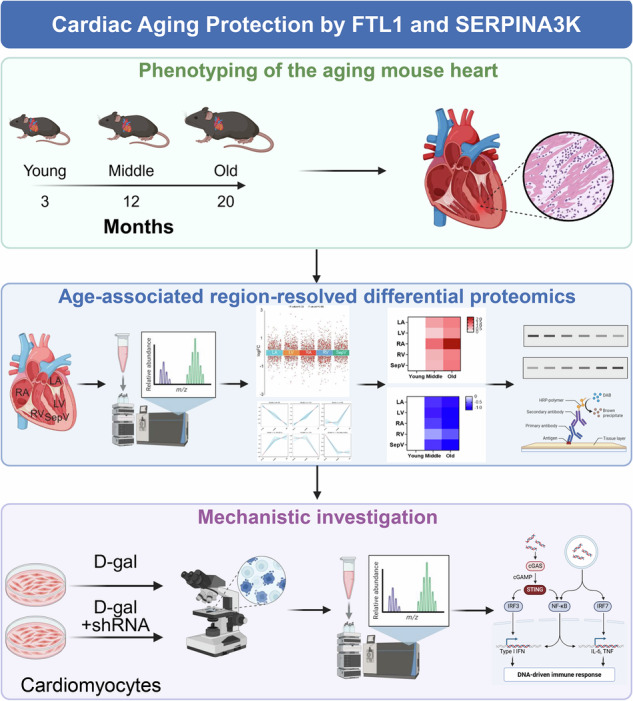

## Introduction

As the primary risk factor for cardiovascular diseases (CVDs), aging drives pathogenesis through structural degeneration, molecular damage, and systemic inflammatory responses [1, 2]. As the central organ of the circulatory system, the heart propels blood through the vascular network to deliver oxygen and nutrients and remove metabolic waste throughout the body. Consequently, aging-induced structural alterations and functional decline in this critical organ significantly elevate vulnerability to cardiac dysfunctions, including heart failure, arrhythmias, and coronary artery disease [3].

The heart is a four-chambered organ (left atrium [LA], left ventricle [LV], right atrium [RA], right ventricle [RV]) performing specialized physiological functions. Cardiac aging entails structural remodeling, progressive functional decline, and concomitant cellular phenotypic alterations [4]. Specifically, aging-related remodeling of the left atrium is characterized by gradual dilation and fibrosis, which contribute to functional deterioration, including impaired reservoir and conduit functions, conduction delays, reduced total emptying fraction, and an increased risk of mortality, primarily due to fibrotic stiffening [5, 6]. Left ventricular remodeling is characterized by myocardial hypertrophy with concomitant cavity reduction, driven by impaired calcium handling, oxidative stress, cardiomyocyte loss, and increased collagen deposition [7, 8]. Alongside this structural adaptation, age-related declines in diastolic function, manifested as impaired relaxation and increased ventricular stiffness, significantly contribute to diastolic dysfunction in the elderly, thereby establishing advanced age as a key predictor for Heart Failure with Preserved Ejection Fraction (HFpEF), where ventricular-arterial stiffening and elevated left ventricular stiffness are identified as core underlying mechanisms [9].

Cardiac tissue comprises a diverse array of cell types, mainly including cardiomyocytes, endothelial cells, fibroblasts, and resident immune cells. Although cardiomyocytes constitute only ~30% of the cardiac cell population, they account for more than 70% of the myocardial mass due to their substantially larger volume compared to non-cardiomyocytes [10, 11]. The integrated pumping function of the heart arises from the coordinated intercellular communication within this dynamic cellular network. However, this communication also contributes to aging-related dysfunction through factors associated with the senescence-associated secretory phenotype (SASP), pro-inflammatory cytokines, and immunomodulatory molecules [12]. These factors contribute to the development of cellular senescence characteristics, including cell cycle arrest, mitochondrial dysfunction, increased oxidative stress, DNA damage, and progressive fibrosis [13]. Consequently, this leads to a self-perpetuating cycle of senescence and chronic inflammation that impedes proliferation, blocks regeneration, causes tissue damage, and culminates in age-related cardiometabolic diseases [14].

The intricate process of cardiac aging is significantly influenced by proteins, which play critical roles in nearly all physiological functions. Therefore, delineating the spatiotemporal dynamics of protein expressions across different regions of the heart throughout the aging process is vital for clarifying the pathogenic mechanisms that connect aging to heart disease and for identifying potential targets for anti-aging pharmacological interventions. Leveraging mass spectrometry (MS)-based proteomics, a robust approach for uncovering the spatiotemporal expression patterns of proteins, previous studies have successfully characterized the regional cardiac proteome in healthy human subjects [11], and explored the regional and cell type-specific proteomes in mice [15], as well as the regional cardiac proteomes across multiple species, including non-human primates like orangutans [16]. Nevertheless, a systematic comparative analysis of the protein compositions and their dynamic changes across different cardiac regions and age groups has yet to be conducted.

In this study, we aimed to construct a spatially resolved proteomic atlas of the mouse heart that encompasses various age demographics across different anatomical regions of the mouse heart. Our region-resolved proteomic mapping uncovers that age-associated cardiac remodeling is the predominant factor, often overshadowing region-specific functional specializations. By employing an integrated bioinformatics approach that combines clustering analysis, functional enrichment, Weighted Gene Co-expression Network Analysis (WGCNA) with Random Forest (RF)-based machine learning, we have successfully identified and validated Ferritin light chain 1 (FTL1) and Serine protease inhibitor A3K (SERPINA3K) as key protein regulators that exhibit significant differential expression across all cardiac regions.

FTL1, the light chain subunit of iron storage protein ferritin, plays a crucial role in the maintenance of cellular iron homeostasis and acts as a key regulator of ferroptosis [17, 18]. Notably, iron accumulation is linked to cellular senescence associated with atherosclerosis, and the overexpression of FTL1 in endothelial cells can reduce labile iron and mitigate senescence [19]. SERPINA3K, a member of the serine protease inhibitor family and a functional ortholog of human SERPINA3, is involved in various pathological mechanisms, including fibrosis, inflammation, and oxidative stress [20]. In the context of cardiac ischemia-reperfusion injury, both the expression and lactylation of SERPINA3K have been implicated in the protection of cardiomyocytes from apoptosis [21]. Nevertheless, the roles of both FTL1 and SERPINA3K in the context of cardiac aging have not been explored yet.

Collectively, we have successfully constructed a regionally resolved proteomic map of cardiac aging, highlighting key protein regulators that may serve as mechanistic targets for strategies aimed at mitigating age-related cardiovascular deteriorations.

## Methods

### Mice

All animal experiments were performed in compliance with the Guide for the Care and Use of Laboratory Animals and approved by the Institutional Animal Care and Use Committee of Shenzhen People’s Hospital (Approval No.: AUP-220302-HJN-026-01). Male C57BL/6 J mice (Charles River Laboratories, Guangzhou, China) were housed under specific pathogen-free (SPF) conditions with a 12-h light/dark cycle. Blood was collected after euthanasia by cervical dislocation. Hearts were subsequently perfused with 10 mL ice-cold phosphate-buffered saline (PBS, 10010023, Gibco, Waltham, MA, USA). For proteomic and western blot (WB) analyses, hearts were dissected into five anatomical regions: LA, LV, RA, RV, and ventricular septum (SepV). Tissues were flash-frozen in liquid nitrogen and stored at -80°C.

### Cell culture

The HL-1 cardiac muscle cell line used in this research was obtained from a commercial vendor (FH1101, Shanghai Fuheng, Shanghai, China). The cells were cultured in DMEM medium (C11995500BT, Gibco) supplemented with 10% FBS (FSP500, ExCell, Suzhou, China) and 1% penicillin-streptomycin (15140122, Gibco) at 37 °C/5% CO_2_. All experiments used cells within 15 passages post-thaw.

### Construction of knockdown and overexpression cell lines

The shRNAs for stable knockdown of *Ftl1* and *Serpina3k* (*Sa3k*) plasmid were designed in the online GPP Web Portal of the Broad Institute and synthesized by Sangon (Shanghai, China). A *Sa3k* overexpression plasmid was obtained from Hanyi Bio (Guangzhou, China). Lentiviral particles were produced by co-transfecting HEK293T/17 cells with the respective shRNA or overexpression plasmids alongside the packaging plasmids psPAX2 and pMD2.G. HL-1 cells were transduced with lentivirus for 48 h, followed by a 7-day selection using 2 μg/mL puromycin (P8230, Solarbio, Beijing, China). The efficiencies of knockdown and overexpression in HL-1 cells were measured by WB analysis. The shRNA sequences are provided in Supplemental Table S1.

### Proteomics sample preparation

Cardiac tissues were homogenized in 500 µL of lysis buffer using a 15-s mechanical disruption step, followed by ice-cold sonication (3 s on, 7 s off) for 1 min. Homogenates were centrifuged (14,000 × *g*, 20 min, 4 °C), and a 10 µL aliquot of the supernatant was reserved for BCA (23225, Thermo Fisher Scientific) protein quantification. The proteomics sample preparations were conducted as previously described (details in Supplemental Methods) [22, 23].

### Liquid chromatography-tandem mass spectrometry (LC-MS/MS) analysis

Peptides were analyzed by LC-MS/MS using an Orbitrap Eclipse Tribrid mass spectrometer equipped with an EASY-nLC 1200 system (both from Thermo Fisher Scientific), as previously described [22, 23]. All LC-MS/MS data were processed using DIA-NN (version 1.8.1) in spectral library-free mode [24] against the Mus musculus proteome, which was downloaded from Swiss-Prot (17 184 entries, Jan 2024). The database search was performed with trypsin specified as the enzyme, allowing up to one missed cleavage. Cysteine carbamidomethylation was set as a fixed modification, while methionine oxidation and N-terminal acetylation were included as variable modifications. A 1% false discovery rate (FDR) was applied at both the peptide and protein levels using a decoy search strategy. Default mass accuracy settings were used for MS1 and MS2, and match-between-runs (MBR) was enabled. The mass spectrometry proteomics data have been deposited to the ProteomeXchange Consortium via the PRIDE [25] partner repository with the dataset identifier PXD066926.

### Differential expression analysis

Differential expression analysis was conducted using the DEP2 package [26], with limma-based empirical Bayes models, including only proteins detected in over 70% of samples. Functional enrichment for Gene Ontology (GO) and KEGG pathways was conducted using clusterProfiler (v4.4.4) and Metascape [27]. Over-representation analysis employed Fisher’s exact test, with *p* values adjusted for multiple comparisons using the Benjamini-Hochberg method.

### Weighted gene co-expression network analysis (WGCNA)

WGCNA was carried out using the WGCNA shiny app https://github.com/ShawnWx2019/WGCNA-shinyApp following the guidelines [28].

### Random forest (RF) modeling

The proteomics dataset was preprocessed by replacing missing values with feature-wise means and removing non-numeric columns. An RF classifier (n_estimators = 75, random_state = 0, n_jobs = -1) was trained to associate protein expression patterns with biological targets. Feature importance was quantified using Gini impurity reduction, with subsequent analysis of importance distribution (max, min, mean, SD) and threshold-based feature counts (0.001–0.01).

### Cell viability assay

Cells were seeded in 96-well plates (500 cells/well). Following adhesion, cultures were induced to an aging state with 10 g/L D-galactose (D-gal, G5388, Sigma-Aldrich) for 48 h. Ferrostatin-1 (Fer-1; HY-100579, MedChemExpress, Princeton, NJ, USA) was used at a 2 μM final concentration to inhibit ferroptosis. Following treatment, cells were incubated with 100 μL CCK-8 (HY-K0301, MedChemExpress) for 1 h at 37 °C, and absorbance was measured at 450 nm using a microplate reader.

### RT-qPCR

Total RNA was isolated with TRIzol reagent (T9424, Sigma-Aldrich). For each reaction, 1 μg RNA was reverse transcribed to cDNA using PrimeScript™ RT Kit (RR037A, Takara, Japan). The qPCR analysis was performed utilizing TB Green® Premix Ex Taq™ II (RR820A, Takara) on an ABI StepOne Plus system, following manufacturer specifications, with amplification specificity verified by melt curve analysis. Relative gene expression 2^(−ΔΔCt) was normalized to β-actin (*Actb*), with primers listed in Supplemental Table S2.

### Western blot (WB)

Protein lysates were prepared in RIPA buffer (P0013B, Beyotime, Shanghai, China) supplemented with protease inhibitors. Samples (30 μg aliquots) were denatured in 5× loading buffer (95 °C, 10 min), separated by 12.5% SDS-PAGE gels, and transferred to PVDF membranes (IPVH00010, Sigma-Aldrich). Membranes were blocked with 2.5% BSA/TBST (1 h, RT), incubated overnight at 4 °C with primary antibodies. Followed by HRP-conjugated secondary antibodies (1 h, RT). Protein bands were visualized using ECL Western blotting substrate (1705061, BioRad, CA, USA) and quantified with ImageJ. All antibodies are cataloged in Supplemental Table S3, and uncropped original blots are included in the Supplemental materials.

### Reactive oxygen species (ROS) quantification

ROS were quantified using a ROS Detection Kit (E-BC-F005, Elabscience®, Wuhan, China). Following 48-h treatment with 10 g/L D-gal, cells were incubated with the ROS-sensitive fluorescent probe Dihydroethidium at 37 °C for 1 h in serum-free medium. Fluorescence images were acquired using an inverted microscope with a Cy3 filter set (Olympus, Japan). Quantitative analysis of mean fluorescence intensity (MFI) was conducted using the ImageJ software.

### Ferroptosis-related assay

For each detection, 5 × 10^6^ cells were collected. Malondialdehyde (MDA) levels were determined by thiobarbituric acid reactive substances (TBARS) assay kit (E-BC-K028-M; Elabscience®), and Fe^2+^ concentration was determined with the Cell Ferrous Iron Fluorometric Assay Kit (E-BC-F101, Elabscience®). GSH concentration was determined with the GSH and GSSG Assay Kit (S0053, Beyotime).

### Senescence-associated β-galactosidase (SA-β-Gal) staining

Cellular senescence was assessed via SA-β-Gal staining using a commercial kit (C0602, Beyotime). Cells were seeded in 12-well plates (3 × 10^4^ cells/well) and subjected to the following treatments after 24 h of adhesion: sh*NC* and sh*Ftl1* cells: 10 g/L D-gal for 48 h, sh*Ftl1*+Fer-1 cells: 10 g/L D-gal + 2 μM Fer-1 for 48 h. Following PBS washes, cells were fixed for 20 min (RT) and stained at 37 °C for 24 h under CO_2_-free conditions. Senescent cells were identified by cytoplasmic blue puncta using bright-field microscopy (Olympus, Japan).

### H&E, Masson's trichrome, and immunohistochemistry (IHC) staining

Fresh cardiac tissues were fixed in 4% paraformaldehyde (PFA; BL539A, Biosharp, Jiangsu, China) at 4 °C for 24 h. Following paraffin embedding and sectioning at 5 μm, histological assessment was performed using Hematoxylin and Eosin (H&E; C0105, Beyotime) to assess general morphology, and Masson’s trichrome (D026, LEAGENE, Beijing, China) to evaluate collagen deposition. Staining procedures were conducted according to manufacturers’ protocols with appropriate controls. Image acquisition was conducted using bright-field microscopy at a magnification of 20×.

IHC analysis was performed on 5-μm FFPE tissue sections. After deparaffinization in xylene and graded ethanol rehydration, antigen retrieval was conducted in EDTA (pH 8.0, ST069, Beyotime) using a pressure cooker (95 °C, 30 min), then blocked with 3% H_2_O_2_ (25 min, RT, dark), followed by 3% BSA blocking (1 h, RT). Primary antibodies were incubated at 4 °C overnight, and secondary antibodies were incubated at RT for 1 h. Detection used 3,3’-diaminobenzidine (DAB, P0202, Beyotime) with hematoxylin counterstaining. The sections were then dehydrated through ethanol/xylene before being coverslipped. Slides were imaged using bright-field microscopy. Antibody specificity was validated via isotype controls as well as omission controls.

### Cytosolic mitochondrial DNA (mtDNA) extraction and quantification

Cytosolic mtDNA was extracted as described [29]. Cell sample aliquots (10^6^ cells each) were divided equally for parallel processing: (1) Total mtDNA normalization control: suspended in 300 μL 50 mM NaOH, boiled (95 °C, 30 min), and neutralized with 30 μL 1 M Tris-HCl (pH 8.0, Beyotime). (2) Cytosolic fraction isolation: permeabilized in 300 μL digitonin buffer (150 mM NaCl, 50 mM HEPES, pH 7.4, 25 μg/mL digitonin; MedChemExpress HY-N4000) with rotation (10 min, RT). Intact cells were pelleted by sequential centrifugation (980 × *g*, 4 °C, 3 min; thrice), followed by centrifugation at 17,000 × *g*, 20 min, 4 °C to remove residual debris. The quantification of mtDNA was performed using quantitative PCR as previously described. Primer sequences are provided in Supplementary Table S2.

### Statistical analysis

Data are presented as mean ± standard deviation (SD) from at least three independent experiments, and all samples and cells were included in the analysis. Group comparisons were performed using unpaired *t* tests performed using GraphPad Prism 8 software (GraphPad Software, CA, USA). Associations between variables were assessed using Spearman correlation analysis. All tests were two-tailed, and statistical significance was defined as *p* < 0.05. Specific numbers of replicates are indicated in the figure legends.

## Results

### Characterization of age-related phenotype changes in the heart and plasma

**Fig. 1:**
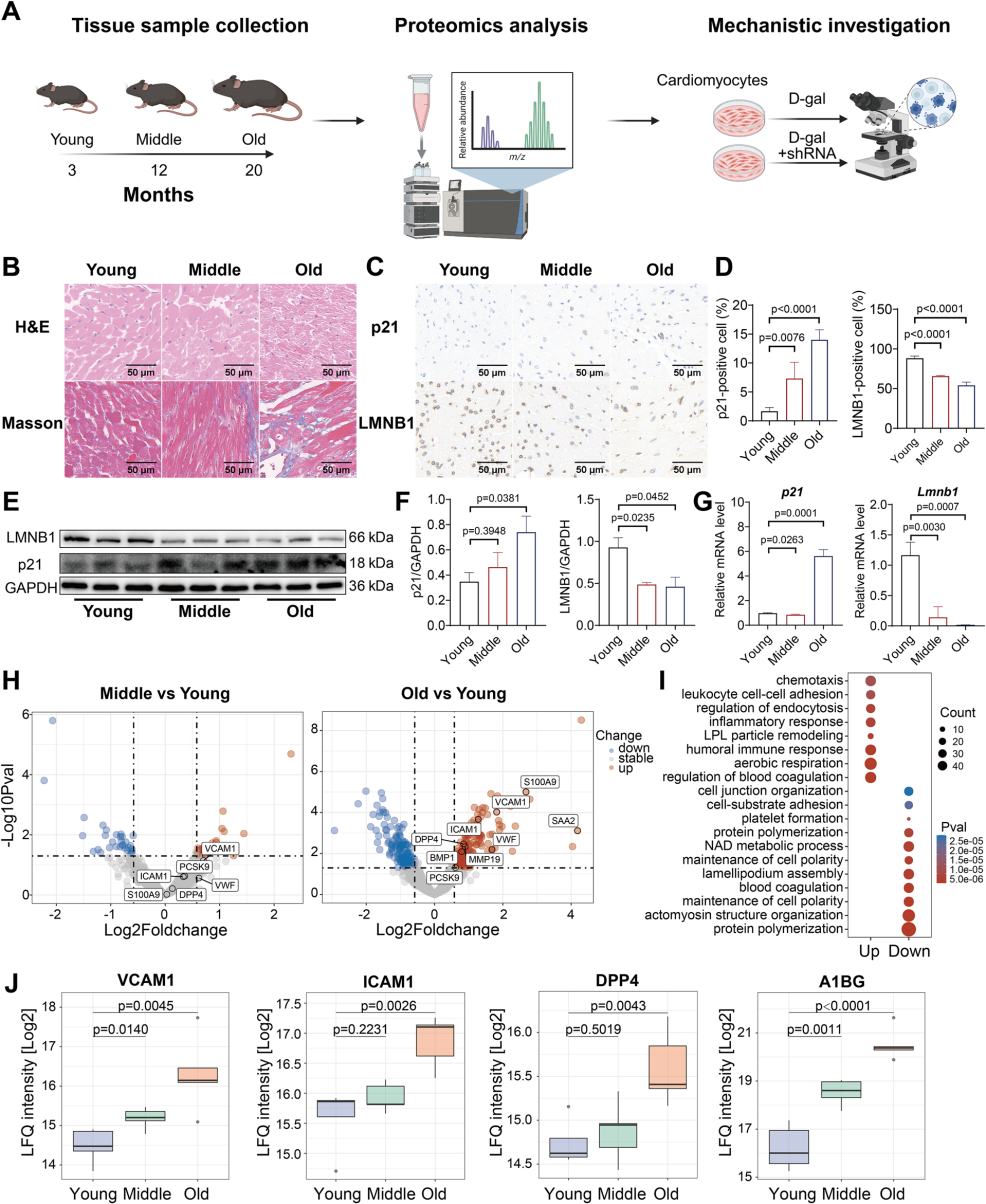
Phenotypical characterization of age-related changes in the mouse heart and plasma. **A** Schematic overview of the experimental workflow, encompassing the collection of murine heart tissues from young (3-month-old), middle-aged (12-month-old), and old (20-month-old) mice, subsequent DIA proteomic profiling, and bioinformatic selection, followed by functional validation of key proteins. Created with BioRender.com. **B** Representative H&E (top) and Masson’s trichrome (bottom) staining of cardiac tissues from the three different age groups of mice (scale bar = 50 μm). **C** IHC staining for biomarkers p21 and LMNB1 in cardiac tissues across different age groups (scale bar = 50 μm). **D** Quantification of the proportions of p21 and LMNB1 positive cells (*n* = 3). **E** Representative Western blots of p21 and LMNB1 in cardiac tissues. **F** Quantitative analysis of p21 and LMNB1 levels normalized to loading controls (GAPDH) (*n* = 3). **G** RT-qPCR analysis of the expression of *p21* and *Lmnb1* mRNA in cardiac tissues (*n* = 3). **H** Volcano plots depicting DEPs in plasma: Middle-aged vs. Young (left) and Old vs. Young (right), with significantly upregulated and downregulated proteins denoted in red and blue, respectively (*n* = 5). **I** GO enrichment analysis of biological processes for the DEPs in plasma in Old vs. Young mice. Node size corresponds to the count of DEPs; color gradient indicates the significance of enrichment. **J** Box plots showing log_2_(LFQ) intensity values of VCAM1, ICAM1, DPP4, and A1BG across the different age groups (*n* = 5). Statistical significance was determined using unpaired t-tests. Replicates are plasma/cardiac tissues from different mice.

We first carried out a series of histological analyses on cardiac tissues obtained from mice belonging to three different age groups: Young (3-month-old), Middle-aged (Middle, 12-month-old), and Old (20-month-old) (Fig. 1A). The staining results indicated that aged mouse hearts exhibited a distinct pattern of age-related cardiac remodeling, characterized by disorganized arrangement of cardiomyocytes, irregular cellular morphology, and progressive fibrosis (Fig. 1B). An evaluation of senescence markers CDKN1A/p21 and LMNB1 in the aging mouse hearts demonstrated a progressive increase in CDKN1A/p21 and a decrease in LMNB1 at both the protein (Fig. 1C–F) and transcriptional (Fig. 1G) levels.

In addition, we performed an in-depth plasma proteomics analysis to examine age-related differentially expressed proteins (DEPs) in plasma protein profiles in mice [30]. This analysis unveiled significantly elevated levels of proteins associated with coagulation cascade signaling and pro-inflammatory mediator pathways in the aged groups (Fig. 1H, I). Notably, we observed a progressive accumulation of biomarkers related to cardiovascular inflammation, including ICAM1, VCAM1, A1BG, DPP4, PCSK9, and S100A9, as age increased (Fig. 1H, J).

These findings indicated that the aging process stimulates the systemic release of various cardiovascular pathophysiological mediators into circulation, thereby possibly contributing to a pro-thrombotic and pro-inflammatory milieu that heightens vulnerability to cardiovascular diseases.

### Region-resolved proteome profiling in mouse hearts of different ages

**Fig. 2:**
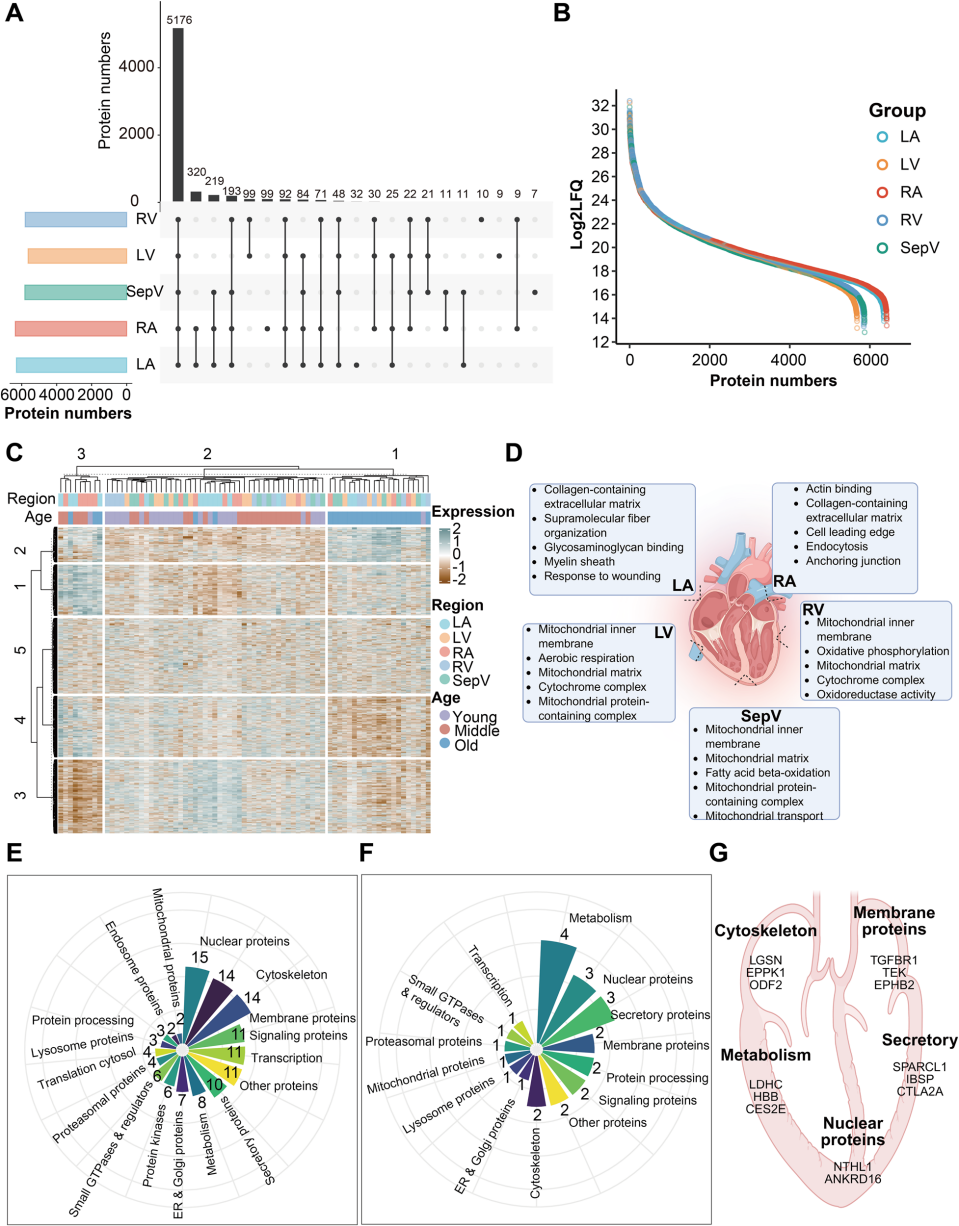
Region-resolved quantitative proteomic profiling of heart tissues. **A** Upset plot illustrating the overlap of protein identification across five distinct cardiac regions: LA, LV, RA, RV, SepV (*n* = 5). **B** The ranges of protein abundances across five cardiac regions are visualized (*n* = 5). **C** Heatmap displaying the protein expression profiles from the different cardiac regions and age groups (*n* = 5 per age and region combination). **D** Region-specific GO biological process enrichment reveals the significantly altered pathways against the global proteomic background (*n* = 5). The rose plot depicts the number of proteins within the functional classification specifically identified in the atria (**E**) or ventricles (**F**), respectively (*n* = 5). **G** The representative proteins that were uniquely identified in each of the five cardiac regions (*n* = 5). Replicates are cardiac tissues from different mice.

Next, we meticulously dissected mouse hearts from different ages into five distinct anatomical regions: LA, LV, RA, RV, and SepV, to perform quantitative proteomic analysis using data-independent acquisition (DIA) methodology. As each age group, specifically 3-month, 12-month, and 20-month-olds, comprised 5 mice, a total of 75 samples were profiled across the different age and region combinations. A total of 6 650 proteins were successfully quantified, with 5 176 (77.8%) being common across all regions (Fig. 2A). The observed range of protein abundance spanned nearly six orders of magnitude (Fig. 2B), highlighting the remarkable depth of this proteomic dataset. Furthermore, an unsupervised clustering analysis of the proteomic expressions revealed distinct protein expression patterns influenced by both cardiac region and age, with age emerging as the more predominant factor (Fig. 2C). In examining the effects of aging, we found that proteomic alterations between young and middle-aged hearts were minimal; however, the hearts from old mice displayed significant divergence, indicating an accelerated aging process later in life (Fig. 2C and Table S4). This finding is consistent with a recent report that the onset of age-related functional decline occurs at around 50 in humans [31].

Despite the predominant influence of age, distinct regional signatures were also discernible. According to protein expression patterns, the ventricles and septum exhibited close clustering, in contrast to the atria (Fig. 2C). Specifically, proteins that were enriched in the LA and RA were largely associated with the collagen-containing extracellular matrix. Those primarily found in the LV, RV, and SepV were notably enriched within mitochondrial functional pathways (Fig. 2D and Table S5). Moreover, we also observed region-specific protein profiles, with the RA, LA, RV, LV, and SepV exhibiting 99, 32, 10, 9, and 7 unique proteins, respectively (Fig. 2A and Table S6). We categorized these proteins into atrial and ventricular groups, respectively. Based on protein function classification [32], we found that proteins identified uniquely in the atria were predominantly nuclear, cytoskeletal, and membrane proteins (Fig. 2E, G), while those specific to the ventricles were primarily metabolic, nuclear, and secreted proteins (Fig. 2F, G), highlight the ventricles’ heightened energetic demands for pumping blood compared to the atria.

### Region-resolved mapping of age-associated proteomic alterations in the mouse heart

**Fig. 3:**
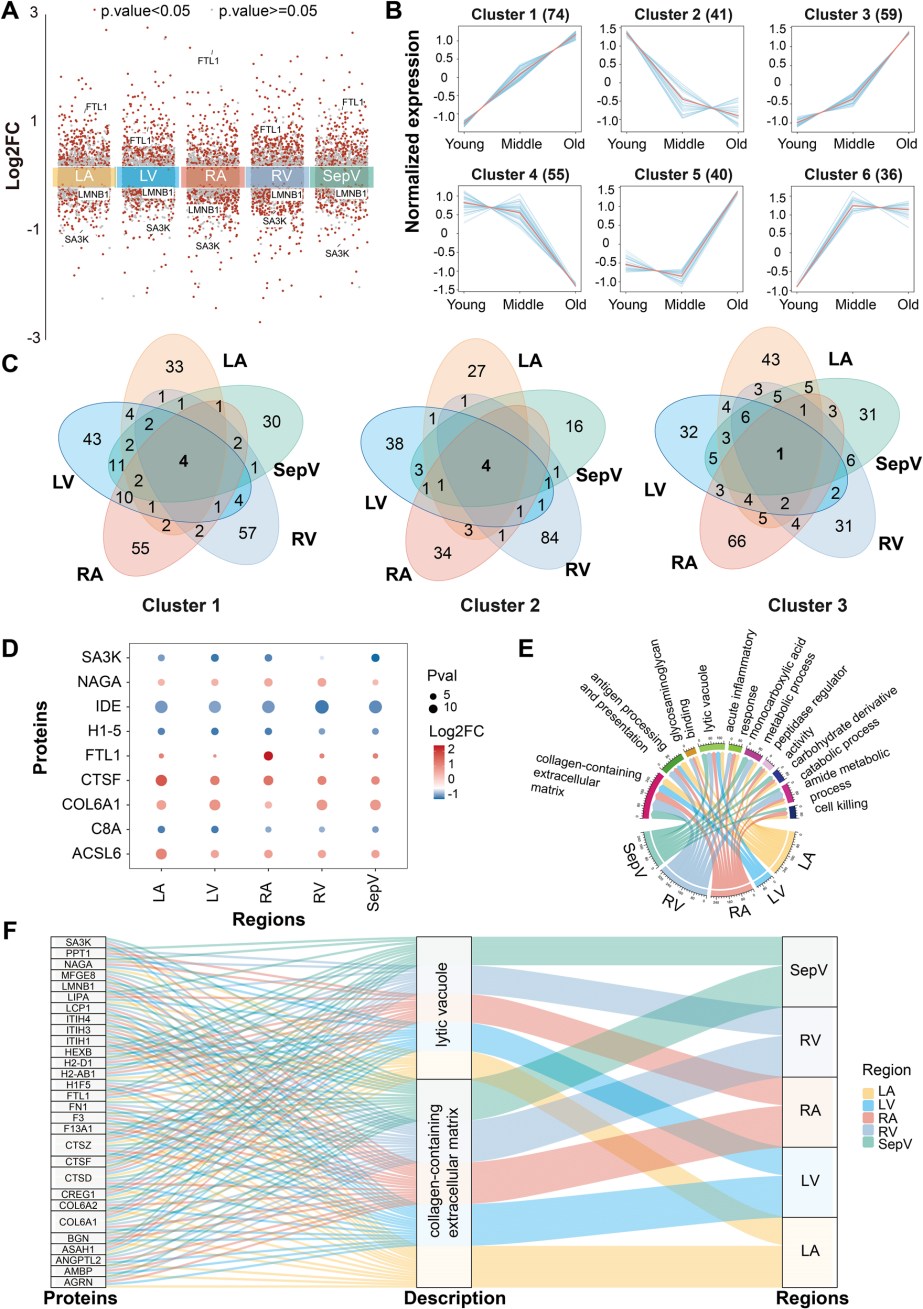
Aging-associated proteome dynamics across region-resolved mouse heart. **A** Multi-group volcano plots illustrate the proteomic changes associated with aging by comparing the hearts of the Old and Young mice across different cardiac regions, with red dots denoting proteins that exhibit significant dysregulation ( | FC | > 1.3, *p* < 0.05) (*n* = 5). **B** Mfuzz clustering illustrates the trajectories of aging-related proteins in the LV, where membership is determined based on z-score-normalized LFQ intensities (*n* = 5). **C** Venn diagrams highlight the dysregulated proteins with conserved expression patterns in different cardiac regions (*n* = 5). **D** The expression profiles of 9 consensus dysregulated proteins are presented, with red color indicating upregulation in aged cohorts and blue color indicating downregulation. The circle area reflects the statistical significance as measured by the *p* value (Pval = -Log10(p value)) (*n* = 5). **E** The Circos plot illustrates the enrichment of region-specific GO biological processes in Old vs. Young groups. Correlation arcs connect enriched pathways to various anatomical regions of the heart: LA (cyan), LV (vermilion), RA (azure), RV (olive), SepV (green) (*n* = 5). **F** Sankey diagram illustrating the pathway-associated proteins that are co-enriched in GO biological processes across the five cardiac regions in aged cohorts (*n* = 5). Statistical significance was performed using an empirical Bayes moderated *t* test. Replicates are cardiac tissues from different mice.

To characterize age-associated proteomic alterations across various cardiac regions, we utilized a multi-group volcano plot to identify DEPs. A comparison between the Old and Young mice revealed substantial changes: specifically, we identified 297 DEPs in the LA; 355 DEPs in the LV; 378 DEPs in the RA; 514 DEPs in the RV; and 312 DEPs in the SepV (Fig. 3A and Table S7). Similarly, in comparison of Middle-aged mice to Young controls, our analysis uncovered 314 DEPs in the LA; 187 DEPs in the LV; 123 DEPs in the RA; 197 DEPs in the RV; and 113 DEPs in the SepV (Fig. S1A, B, and Table S7).

Subsequently, we categorized the DEPs according to their expression patterns for each specific cardiac region. Our analysis indicated that a substantial proportion of the DEPs can be segregated into six distinct clusters that reflect various age-related changes within each region. Notably, while the overall clustering patterns were largely similar across the different cardiac regions, some unique patterns emerged. Among these identified clusters, three clusters exhibited consistent expression patterns across all five cardiac regions and thus were consistently labeled as Cluster 1, Cluster 2, and Cluster 3 in the cluster classifications for each region (Fig. 3B and Fig. S1C–F). Following the clustering analysis, we next focused on the DEPs that displayed conserved expression patterns across the cardiac regions, resulting in the identification of 9 proteins, including ACSL6, C8A, COL6A1, CTSF, FTL1, H1-5, IDE, NAGA, and SERPINA3K (SA3K) (Fig. 3C, D).

Furthermore, we conducted a functional enrichment analysis on all DEPs identified in the hearts of aged mice. GO enrichment analysis revealed that the pathways significantly enriched across all five cardiac regions included “collagen-containing extracellular matrix” and “lytic vacuole”, with 29 proteins common to both pathways present in all regions (Fig. 3E, F). KEGG enrichment analysis identified significant pathways, including “complement and coagulation cascades”, “ferroptosis”, and “lysosome”, which were enriched across all five regions, with 19 proteins common to all three pathways in every cardiac region (Fig. S1G, H).

### FTL1 and SA3K were identified and validated as key regulators of cardiac aging

**Fig. 4:**
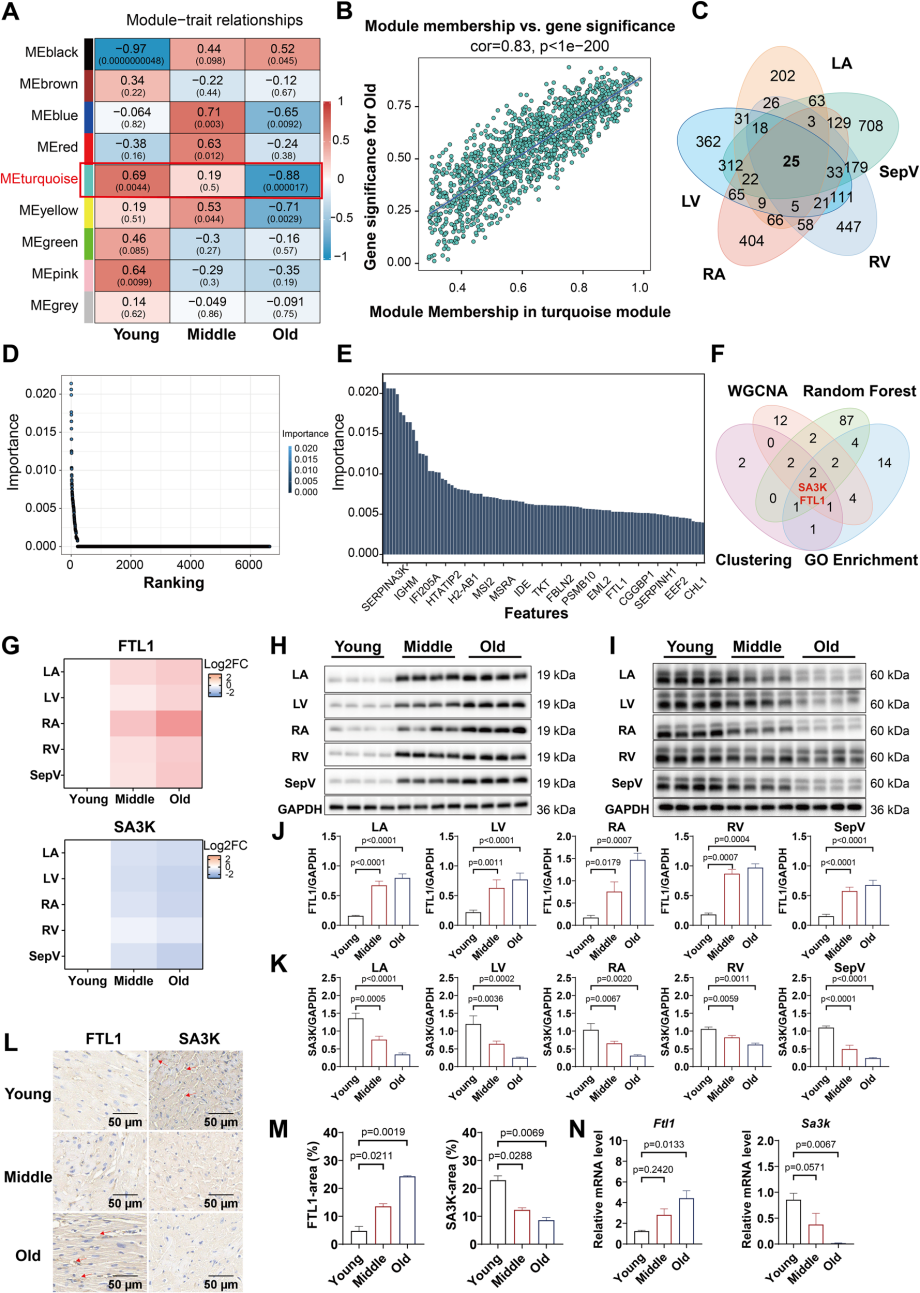
FTL1 and SERPINA3K (SA3K) were identified as key regulators of cardiac aging. **A** Heatmap visualization of the module-trait associations in the LV region. The values displayed indicated the correlation coefficients (upper values) and statistical significance (*p* values; lower values) between module eigengenes (rows) and cardiac aging (columns) (*n* = 5). **B** The scatter plot depicts the correlation analysis with aging for the module most significantly associated with aging (*n* = 5). **C** The Venn diagram illustrates the overlap of aging-associated proteins chosen by WGCNA across five distinct cardiac regions (*n* = 5). **D** The distribution of importance scores for all the quantified proteins in association with cardiac aging, as analyzed by an RF model (*n* = 5). **E** The distribution of importance scores for the top 2% biomarker proteins related to cardiac aging as revealed by RF model analysis (*n* = 5). **F** Key consensus proteins identified through four independent approaches, including clustering analysis, functional enrichment, WGCNA, and RF-based machine learning, were integrated and visualized in a Venn diagram (*n* = 5). **G** Heatmap displays the relative level changes of FTL1 and SA3K proteins in different heart regions during aging (*n* = 5). **H** The WB analysis shows the protein expression of FTL1 in different age groups across five cardiac regions. **I** The WB analysis shows the protein expression of SA3K in different age groups across five cardiac regions. Quantitative analysis quantified the normalized FTL1 (**J**) and SA3K (**K**) levels relative to loading controls (GAPDH) (*n* = 4). **L** IHC staining for FTL1 and SA3K in cardiac tissues across age groups (scale bars = 50 μm), with the red arrow pointing to the changing area. **M** Quantification of the proportions of FTL1 and SA3K positive area (*n* = 3). **N** RT-qPCR assessed the mRNA expression dynamics of *Ftl1* and *Sa3k* (*n* = 3). Statistical significance was performed using unpaired *t*tests. Replicates are cardiac tissues from different mice.

To uncover the critical proteins associated with cardiac aging, we performed a WGCNA to pinpoint the principal protein modules associated with aging. In the LV region, we identified nine distinct protein co-expression modules (Fig. 4A and Table S7). Among these, the turquoise module exhibited strong correlations with both the Young and Old groups (Fig. 4A, B). A similar pattern was also observed for the turquoise module in the RV and SepV; while in the LA, the corresponding module was classified as blue. In the RA, both the blue and green modules displayed similar expression patterns and strong correlations with both the Young and Old groups (Figure S2A-I). Ultimately, we identified 25 core proteins that are closely associated with aging within these consistently correlated modules (Fig. 4C). Additionally, we employed a RF-based machine learning approach to screen the quantified 6 650 proteins and selected the top 2% most associated with cardiac aging (Fig. 4D, E). Finally, an integrative analysis combining results from clustering analysis, functional enrichment, WGCNA, and machine learning led to the identification of FTL1 and SA3K as pivotal proteins that demonstrated age-dependent changes across all cardiac regions (Fig. 4F and Table S7).

Our proteomic profiling indicated an obvious increase in FTL1 expression and a decrease in SA3K expression with advancing age across five distinct cardiac regions (Fig. 4G). These expression changes were further validated within murine cardiac tissue through WB and IHC analysis (Fig. 4H–M and Fig. S2J, K). Consistently, quantitative reverse transcription polymerase chain reaction (RT-qPCR) analysis demonstrated increased *Ftl1* mRNA expression and decreased *Sa3k* mRNA expression in correlation with increasing age (Fig. 4N).

Collectively, these results position FTL1 and SA3K as potential regulatory factors of cardiac aging in murine models, with their spatially coherent expression and age-dependent dysregulation observed across all cardiac regions.

### FTL1 downregulation promotes cardiomyocyte senescence through ferroptosis

**Fig. 5:**
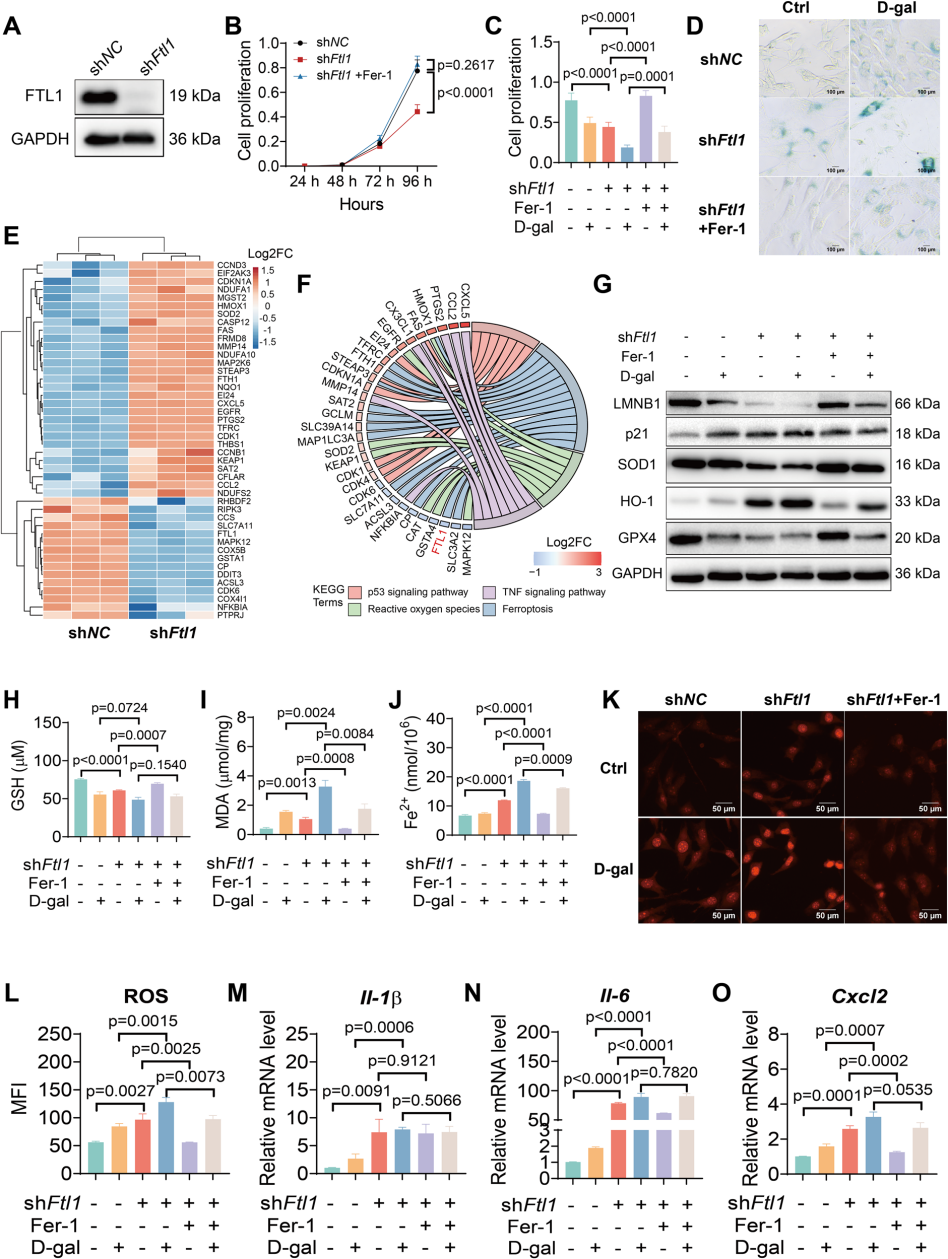
FTL1 is essential for the prevention of cardiomyocyte senescence and ferroptosis. **A** WB analysis confirmed a decrease in FTL1 expression following targeted knockdown. **B** Proliferation analysis revealed that stable sh*Ftl1* cells displayed significantly reduced growth rates compared to the negative control (sh*NC*) (*n* = 6). **C** sh*Ftl1* cells exhibited a more pronounced decline in proliferation following a 48-h treatment with 10 g/L D-gal, which was partially rescued by co-treatment with 2 μM Fer-1 (*n* = 6). **D** SA-β-Gal staining revealed an elevated cellular senescence in sh*Ftl1* cells, which was attenuated by co-treatment with Fer-1 (scale bar = 100 μm). **E** Heatmap displayed DEPs ( | FC | > 1.5, *p* < 0.05) in the sh*Ftl1* cell, with red denoting upregulated proteins and blue indicating downregulated proteins. **F** Circos visualization linking the DEPs (red indicating upregulation; blue indicating downregulation) in sh*Ftl1* versus sh*NC* to key KEGG signaling pathways. **G** WB analysis of p21, LMNB1, HO-1, and GPX4 levels in sh*Ftl1* cells following D-gal treatment, with or without co-treatment with Fer-1. **H-J** Biochemical analyses were conducted to quantify the levels of GSH (**H**), malondialdehyde (MDA) (**I**), and Fe^2+^ (**J**) (*n* = 3). **K** Representative fluorescence staining illustrated the elevated ROS levels in sh*Ftl1* cells, which were reduced following Fer-1 treatment (scale bar = 50 μm). **L** Biochemical analyses were conducted to measure ROS levels (*n* = 3). Transcriptional changes in mRNA expression for *Il-1β* (**M**), *Il-6* (**N**), and *Cxcl2* (**O**) were evaluated by RT-qPCR (*n* = 3). Statistical significance was performed using unpaired *t* tests. Replicates are cells from different cell culture plates.

To investigate the function of FTL1 in cardiac aging, we established a model of shRNA-mediated knockdown in murine HL-1 cardiomyocytes. WB analysis confirmed the effective reduction of FTL1 protein (Fig. 5A and Fig. S3A). Notably, the knockdown of *Ftl1* (sh*Ftl1*) led to marked impairment in cellular proliferation, which could be reversed by treatment with the potent and selective ferroptosis inhibitor Ferrostatin-1 (Fer-1) (Fig. 5B). To establish a model of cardiomyocyte senescence, we treated HL-1 cells with 10 g/L D-gal for 48 h, resulting in a significant reduction in cellular proliferation comparable to the effects observed following *Ftl1* knockdown. Co-treatment with Fer-1 also effectively restored cardiomyocyte proliferation rates compromised by both *Ftl1* depletion and D-gal-induced senescence (Fig. 5C). In addition to the proliferation impairment, SA-β-Gal staining revealed that the depletion of *Ftl1* significantly increased markers indicative of cellular senescence. This pro-senescent effect was effectively counteracted by co-treatment with 2 μM Fer-1 (Fig. 5D).

Proteomic analysis of sh*Ftl1* cells revealed that DEPs were significantly enriched in critical pathways, including p53 signaling, TNF signaling, ROS metabolism, and ferroptosis (Fig. 5E, F, and Fig. S3C–G). *Ftl1* depletion elevated the senescence marker p21 while reducing LMNB1 levels; these changes were effectively reversed by Fer-1 treatment (Fig. 5G and Fig. S3B). Furthermore, *Ftl1* deficiency triggered ferroptosis, as evidenced by diminished expression of key ferroptosis inhibitory protein phospholipid hydroperoxide glutathione peroxidase GPX4 and antioxidant regulator superoxide dismutase [Cu-Zn] (SOD1), accompanied by an increase in the stress-responsive protein heme oxygenase 1 (HMOX1, HO-1). (Fig. 5G and Fig. S3B). Importantly, these ferroptotic alterations induced by *Ftl1* depletion were effectively reverted by co-treatment with Fer-1. This rescue effect was further supported by the concomitant normalization of key markers associated with ferroptosis, including decreased glutathione (GSH) levels, increased malondialdehyde (MDA) accumulation, and elevated Fe^2+^ concentrations (Fig. 5H–J). Fluorescence imaging revealed that *Ftl1* knockdown significantly raised ROS levels, which were effectively mitigated by Fer-1, restoring ROS levels to those observed in the control cells (Fig. 5K, L). Moreover, RT-qPCR analysis demonstrated that *Ftl1* knockdown led to a significant upregulation of various SASP factors, including pro-inflammatory cytokines IL-1β, IL-6, and CXCL2 (Fig. 5M–O). The inflammatory response induced by *Ftl1* depletion was effectively alleviated with Fer-1 treatment.

Collectively, these results underscore the critical role of FTL1 in the protection against cardiac aging. The knockout of *Ftl1* leads to disruption in iron homeostasis, which results in elevated levels of Fe^2+^. This elevation subsequently drives the accumulation of ROS, initiates lipid peroxidation and ferroptosis, induces the release of SASP factors, and ultimately results in cellular senescence.

### SA3K downregulation promotes cardiomyocyte senescence through cGAS-STING-PERK signaling axis

**Fig. 6:**
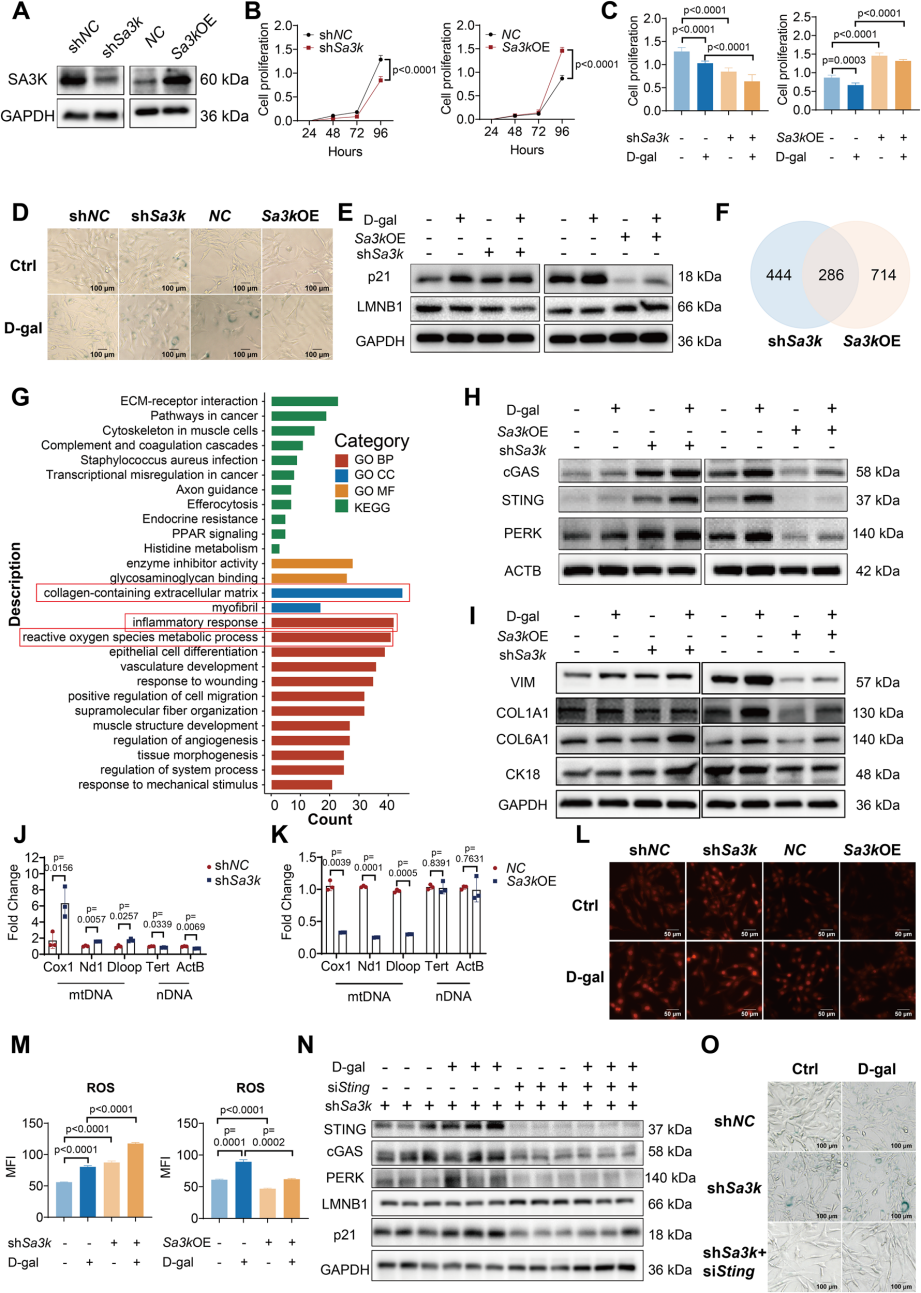
SA3K plays a critical role in the regulation of cardiomyocyte senescence by modulating the cGAS-STING-PERK axis. **A** WB analysis validated the successful depletion of *Sa3k* following shRNA-based knockdown (sh*Sa3k*) and the ectopic overexpression of *Sa3k* (*Sa3k*OE). **B** Proliferation analysis revealed a marked reduction in the growth rates of sh*Sa3k* cells when compared to the negative control (sh*NC*) (left), while *Sa3k*OE cells exhibited enhanced proliferation relative to the control (*NC*) (right), (*n* = 6). **C** Following exposure to a 48-h D-gal (10 g/L) treatment, sh*Sa3k* cells exhibited a pronounced impairment in proliferation (left), while *Sa3k*OE showed resistance to the D-gal-induced growth inhibition (right), (*n* = 6). **D** SA-β-Gal staining revealed a significant increase in senescence markers in sh*Sa3k* cells, while senescence levels were notably reduced in *Sa3k*OE cells (scale bar = 100 μm). **E** WB analysis of p21 and LMNB1 expression in sh*Sa3k* cells and *Sa3k*OE cells. **F** Venn diagram displayed the overlap of DEPs ( | FC | > 1.5, *p* < 0.05) identified in sh*Sa3k* vs. sh*NC* and *Sa3k*OE vs. *NC*. **G** Functional enrichment analysis linked these DEPs to significant pathways (GO BP in red; GO CC in blue; GO MF in orange; KEGG in green). **H** WB analysis of the proteins in the cGAS-STING-PERK axis in sh*Sa3k* cells and *Sa3k*OE cells. **I** WB analysis of the fibrosis-related proteins, including VIM, COL1A1, COL6A1, and CK18, in sh*Sa3k* cells and *Sa3k*OE cells. Barplot showed the quantification of cytosolic mtDNA and nDNA in sh*Sa3k* cells (**J**), and *Sa3k*OE cells (**K**) (*n* = 3). **L** Fluorescence staining revealed elevated ROS levels in sh*Sa3k*, whereas *Sa3k*OE cells exhibited reduced ROS levels (scale bar = 50 μm). **M** Quantitative analysis of changes in ROS production in sh*Sa3k* cells and *Sa3k*OE cells in relation to their respective controls (*n* = 3). **N** WB analysis of all three pathway components following the si*Sting* treatment in sh*Sa3k* cells. **O** SA-β-Gal staining revealed a significant decrease in senescence markers in si*Sting-*treated cells (scale bar = 100 μm). Statistical significance was performed using unpaired *t* tests. Replicates are cells from different cell culture plates.

To investigate the functional role of SA3K in cardiac aging, we first established stable cell lines with either shRNA-mediated depletion of *Sa3k* (sh*Sa3k*) or overexpression of *Sa3k* (*Sa3k*OE), which were verified through WB analysis (Fig. 6A and Fig. S4A). Functional assessments indicated that knockdown of *Sa3k* significantly impaired cardiomyocyte proliferation, while the overexpression of *Sa3k* resulted in a marked increase in proliferation (Fig. 6B). In addition, in a D-gal-induced cellular senescence model, *Sa3k* depletion exacerbated proliferation, whereas its overexpression provided a degree of resistance to D-gal, resulting in minimal impact on proliferation (Fig. 6C). Furthermore, SA-β-Gal staining revealed that *Sa3k* depletion markedly increased cellular senescence, while *Sa3k* overexpression noticeably mitigated senescence levels (Fig. 6D). This observation was supported by WB analysis of senescence-associated markers; sh*Sa3k* cells exhibited elevated expression of p21 and decreased expression of LMNB1, while *Sa3k*OE cells showed reduced p21 levels and elevated LMNB1 expression (Fig. 6E and Fig. S4B).

To further illuminate the molecular mechanisms through which SA3K influences cellular senescence, quantitative proteomic profiling was performed on both sh*Sa3k* and *Sa3k*OE cardiomyocytes (Fig. S4C–F). The analysis revealed that DEPs arising from both genetic manipulations were notably enriched in pathways related to ECM-receptor interaction, inflammatory response, and ROS metabolism (Fig. 6F, G, and Fig. S4G). Importantly, the proteomic analysis identified STING (Stimulator of interferon genes protein) as a critical regulatory node; the depletion of *Sa3k* led to a notable upregulation of STING, while its overexpression resulted in downregulation of STING (Fig. S4E, F). This finding was further validated by WB analysis, showing that *Sa3k* depletion significantly enhanced protein expression within the cGAS-STING-PERK signaling axis, while *Sa3k* overexpression suppressed its activation (Fig. 6H and Fig. S5A–C). Building upon prior evidence that links the cGAS-STING-PERK axis to the regulation of fibrotic processes [33], we demonstrated that sh*Sa3k* significantly upregulates key pro-fibrotic markers, including Vimentin (VIM), Collagen alpha-1(VI) chain (COL6A1), Cytokeratin 18 (CK18), and Collagen alpha-1(I) chain (COL1A1), whereas *Sa3k* overexpression led to a decrease in their expressions (Fig. 6I and Fig. S5D–G).

To investigate the role of SA3K in the activation of the cGAS-STING-PERK axis, the release of intracellular mitochondrial DNA (mtDNA) was quantified. RT-qPCR analysis revealed a dramatic increase in mtDNA levels (including *Cox1*, *Nd1*, and *D-loop*) in sh*Sa3k* cells, alongside a decline in nuclear DNA (nDNA) markers (*Tert* and *Actb*) (Fig. 6J). Conversely, *Sa3k*OE cells exhibited significantly lower mtDNA content without significant alterations in nDNA levels (Fig. 6K). Moreover, *Sa3k* depletion resulted in a marked increase in intracellular ROS levels in cardiomyocytes, while *Sa3k* overexpression led to a decrease in ROS accumulation (Fig. 6L, M). To verify that SA3K indeed regulates cellular senescence through the cGAS-STING-PERK axis, we performed siRNA-mediated knockdown of *Sting* in the sh*Sa3k* HL-1 cell line. The experimental results indicated that the knockdown of *Sting* led to a reduction in the expression of proteins within the cGAS-STING-PERK axis and decreased cellular senescence in sh*Sa3k* cells (Fig. 6N, O and Fig. S5H–L).

Collectively, these results demonstrate that SA3K plays a crucial role in regulating cardiomyocyte senescence and fibrosis through its modulation of the cGAS-STING-PERK axis.

### SA3K attenuates cardiac aging via the cGAS-STING-PERK axis in vivo

**Fig. 7:**
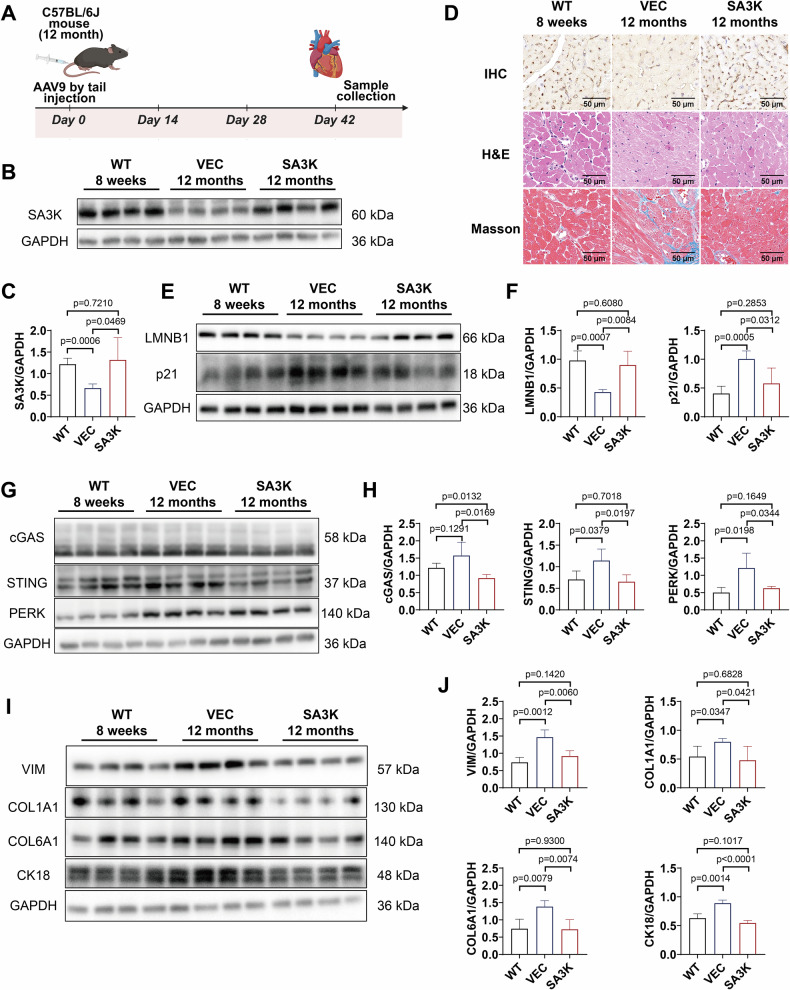
SA3K plays a critical role in the regulation of cardiac aging in vivo. **A** Experimental design to assess the role of SA3K in cardiac aging. 12-month-old mice were tail-injected with either an empty vector (VEC) or an AAV9 construct expressing *Sa3k* (SA3K) for 6 weeks. Created with BioRender.com. **B** WB validated the successful overexpression of SA3K in the heart. **C** Quantitative analysis quantified the normalized SA3K levels relative to loading controls (GAPDH) (*n* = 4). **D** Representative IHC (top), H&E (middle), and Masson’s trichrome (bottom) staining of mouse cardiac tissues from different age groups (scale bars = 50 μm). **E** WB analysis for p21 and LMNB1 in cardiac tissues. **F** Quantitative analysis quantified the normalized p21 and LMNB1 levels relative to loading controls (*n* = 4). **G** WB analysis indicated that SA3K influenced the cGAS-STING-PERK axis in the mouse heart. **H** Quantitative analysis quantified the normalized cGAS-STING-PERK levels relative to loading controls (*n* = 4). **I** WB analysis indicated that SA3K influenced the expression of fibrosis proteins, including VIM, COL1A1, COL6A1, and CK18, with quantitative information in (**J**) (*n* = 4). Statistical significance was determined using unpaired *t* tests. Replicates are heart tissues from different mice.

To further validate the role of SA3K in cardiac aging in vivo, we generated an adeno-associated virus serotype 9 (AAV9) that expresses SA3K under the cardiomyocyte-specific promoter cardiac troponin T (cTnT). Twelve-month-old mice were injected with either an empty vector or an SA3K-expressing virus, while eight-week-old mice were also included as a baseline reference control (Fig. 7A). After six weeks, we evaluated the conditions of the mice. The results indicated that in mice with cardiomyocyte-specific overexpression of SA3K, there was a significant reduction in cardiac fibrosis, and the healthy state of cardiac cells was markedly improved (Fig. 7B–D). Western blot analysis revealed a substantial alleviation of cardiac aging in mice overexpressing SA3K (Fig. 7E, F). Furthermore, we observed that elevated SA3K expression led to significant changes in the protein levels of the cGAS-STING-PERK axis (Fig. 7G, H), along with a marked decrease in the expression of cardiac fibrosis-related proteins (Fig. 7I, J). These results demonstrate that SA3K ameliorates cardiac aging in mice through the cGAS-STING-PERK axis.

## Discussion

MS-based proteomics represents the gold standard for the characterization of protein signatures in multiple fields, including research on aging and CVDs [34–36]. The development of spatial or region-resolved proteomics analysis now enables high-resolution mapping of spatiotemporal proteome dynamics within complex organs. This advancement is exemplified by the generation of a 16-region proteomic atlas of the healthy human heart, establishing a foundational reference for cardiac biology [11]. More recently, studies employing regionally resolved proteomics on human specimens of dilated cardiomyopathy (DCM) have revealed dysregulation in retinoic acid biosynthesis pathways, predominantly in the LV region [15].

The comprehensive region-resolved cardiac proteomic atlas presented in this study delineates the molecular trajectories associated with aging across various murine myocardial regions from 3 to 20 months. This work unveils critical mechanisms underlying cardiac aging and serves as a valuable resource for future mechanistic and translational studies. Notably, we found that the proteomes of the atria are primarily involved in ECM remodeling, while the proteomes of the ventricles are essential for sustaining mitochondrial homeostasis (Fig. 2). Additionally, the regional proteomic analysis enabled the identification of several region-specific proteins: LDHC (L-lactate dehydrogenase C chain), which is reported to be upregulated associated with LV hypertrophy, was exclusively detected in the LV region [37]; SPARCL1 (SPARC-like protein 1), a marker of maladaptive RV remodeling in pulmonary hypertension, was specific to the RV [38]; and TGFBR1 (TGF-beta receptor type-1), associated with atrial fibrillation (AF), was uniquely identified in the RA [39].

Through proteomic profiling, we identified 58 proteins that exhibited significant age-dependent expression changes across all five cardiac regions (Figure S6A and Table S8). A comparative analysis of these proteins with existing literature revealed that 49 of them have been previously linked to aging, 49 to cardiac disease, and 21 specifically to cardiac aging (Fig. S6B–D and Table S8). This high degree of overlap underscores the robustness of our analysis in identifying bona fide cardiac aging-related proteins, many of which are also implicated in the pathogenesis of CVDs.

Leveraging integrated bioinformatics and machine learning frameworks, we have identified FTL1 and SA3K as previously unrecognized key regulators involved in cardiac aging. We note that cross-referencing with the Aging Atlas database (https://ngdc.cncb.ac.cn/aging/index) [40] revealed that there have been no prior reports linking these two proteins to cardiac aging or elucidating their mechanisms of action within this context.

FTL1 is an indispensable structural and functional subunit of ferritin, which is essential for maintaining iron homeostasis [41]. Analysis of Tabula Muris Senis single-cell transcriptomic atlas (https://tabula-muris.sf.czbiohub.org/visualizations) [42] indicated the broad expression of *Ftl1* across multiple cell types in the heart, suggesting its essential role in cardiac functions (Fig. S7). Comparative analysis of several publicly available human transcriptomic datasets revealed a close association between FTL1 expression and aging-related CVDs. Specifically, significantly elevated FTL1 levels were observed in patients of myocardial fibrosis (GSE246298), atrial fibrillation (GSE306533, GSE245886), and aortic valve disease (GSE148219) (Fig. S6E). Moreover, dysfunction of FTL1 has been implicated in the development of preeclampsia and sepsis-induced cardiac injury, primarily due to the accumulation of redox-active iron [43, 44]. Using a D-gal-induced senescent cardiomyocyte model, we demonstrated that *Ftl1* knockdown exacerbates cellular senescence. Mechanistically, FTL1 deficiency promotes ferroptosis and senescence via reduced GPX4 levels, increased ROS, and enhanced SASP, which can be reversed by the inhibition of ferroptosis. It has been documented that with advancing age, iron accumulates in cardiac tissues, which contributes to the development of fibrosis, cellular senescence, and SASP [45]. In response to iron overload, there is an increase in the polysome association and translation of *Ftl1* mRNAs, resulting in elevated expression of FTL1 [46]. In summary, these findings suggest that the upregulation of FTL1 might be an adaptive, compensatory response to protect the heart from age-related oxidative and metabolic stress. One potential mechanism for this protective effect could involve the sequestration of excess Fe^2+^ to suppress ferroptosis.

SA3K is recognized as an acute-phase response marker during acute injuries like cardiac ischemia-reperfusion [21]. The Tabula Muris Senis single-cell transcriptomic atlas indicated that *Sa3k* expression is broadly expressed across diverse cardiac cell types, with the highest mean expression level found in cardiomyocytes (Fig. S8). Analysis of several human aging-associated cardiac disease datasets revealed that SERPINA3 expression is reduced in conditions such as DCM (GSE245825) and aortic valve disease (GSE148219) (Fig. S6F). Furthermore, studies have observed reduced expression of SA3K in the hearts of rat models with type 2 diabetes [47]. Additionally, recent studies have revealed that SA3K protects cardiomyocytes from ischemia-reperfusion-induced apoptosis by inhibiting the pro-apoptotic WNT pathway and activation of the cardioprotective RISK (Reperfusion Injury Salvage Kinase) and SAFE (Survivor Activating Factor Enhancement) pathways [21]. Despite progress in understanding SA3K, its physiological and pathophysiological roles remain incompletely understood.

Here, we report an age-dependent downregulation of SA3K in plasma and cardiac tissues (Figs. 1, 3), and demonstrate that *Sa3k* knockout induces cardiomyocyte senescence, increased ROS production, inflammation, and fibrosis, all of which can be reversed by *Sa3k* overexpression. Mechanistically, *Sa3k* depletion upregulated the expressions of cGAS, STING, and PERK proteins, leading to a senescence phenotype, which was rescued by concurrent *Sting* knockdown (Fig. 6N, O). Conversely, *Sa3k* overexpression suppressed the activation of these factors. These data suggest that SA3K modulates cardiomyocyte senescence, at least in part, through the cGAS-STING-PERK axis. The cGAS-STING-PERK signaling axis serves as the primary sensor for cytosolic dsDNA: during senescence, cytosolic mtDNA activates cGAS to generate cyclic GMP-AMP (cGAMP) from ATP/GTP [48]. cGAMP binding induces STING conformational activation, recruiting PERK via the interaction between STING’s C-terminal tail and PERK kinase domain [29, 33]. We demonstrate that *Sa3k* depletion promotes mtDNA leakage into the cytosol. Thus, the loss of SA3K drives cellular senescence by facilitating mtDNA-dependent activation of the cGAS-STING-PERK axis. To further verify its functional role in vivo, SA3K was specifically overexpressed in cardiomyocytes of 12-month-old mice. The results demonstrate that elevating cardiomyocyte SA3K levels effectively ameliorates cardiac aging phenotypes and reduces associated myocardial fibrosis, potentially through modulation of the cGAS-STING-PERK axis (Fig. 7).

In this study, the main analyses and functional validations have been on the aging-related proteins that are commonly altered across all regions. The rationale behind this was to identify systemic factors of cardiac aging that operate across the entire heart, as these represent the most promising therapeutic targets for addressing age-related cardiac decline. Prioritizing these common factors would allow us to concentrate validation efforts on targets with broad impact potential, thereby enhancing the robustness and generalizability of our findings. In addition, our region-resolved design provided advantages over whole-heart averaged proteomics, which can only capture averaged signals and may risk overlooking important changes. Indeed, we demonstrated that FTL1 and SA3K are consistently dysregulated in every anatomical region, underscoring their critical role in cardiac aging. Nevertheless, future research will explore region-specific key factors and their associated molecular mechanisms.

In conclusion, our region-resolved proteomic atlas of cardiac aging reveals spatiotemporal mechanisms of aging and systematically identifies the key regulators of cardiac aging. Functional validation experiments demonstrated the protective roles of FTL1 and SA3K on aging-related phenotypes. These results also underscore the analytical robustness of this work, positioning our proteomic atlas as a critical resource for investigating region-specific cardiac aging and related pathophysiology. Collectively, this work enhances the understanding of cardiac aging, thereby paving the way for the development of therapeutics against age-related cardiovascular disorders.

## Supplementary information

supplemental material

Table S4

Table S5

Table S6

Table S7

Table S8

uncropped WB figures

## Data Availability

All data generated or analyzed during this study were included in this published article and its supplementary information files.

## References

[1] Obas V, Vasan RS. The aging heart. Clin Sci (Lond). 2018;132:1367–82. 29986877 10.1042/CS20171156

[2] Liu T, Zou J, Geng Q, Liu J. Advances in cardiac telerehabilitation for older adults in the digital age: a narrative review. Heart Mind. 2024;9:321–27.

[3] Xie S, Xu SC, Deng W, Tang Q. Metabolic landscape in cardiac aging: insights into molecular biology and therapeutic implications. Signal Transduct Target Ther. 2023;8:114. 36918543 10.1038/s41392-023-01378-8 PMC10015017

[4] Chen MS, Lee RT, Garbern JC. Senescence mechanisms and targets in the heart. Cardiovasc Res. 2022;118:1173–87. 33963378 10.1093/cvr/cvab161 PMC8953446

[5] Stefani LD, Trivedi SJ, Ferkh A, Altman M, Thomas L. Changes in left atrial phasic strain and mechanical dispersion: effects of age and gender. Echocardiography. 2021;38:417–26. 33594734 10.1111/echo.14997

[6] McManus DD, Xanthakis V, Sullivan LM, Zachariah J, Aragam J, Larson MG, et al. Longitudinal tracking of left atrial diameter over the adult life course: Clinical correlates in the community. Circulation. 2010;121:667–74. 20100973 10.1161/CIRCULATIONAHA.109.885806 PMC2823068

[7] Ozcebe SG, Zorlutuna P. In need of age-appropriate cardiac models: Impact of cell age on extracellular matrix therapy outcomes. Aging Cell. 2023;22:e13966. 37803909 10.1111/acel.13966 PMC10652343

[8] Li H, Hastings MH, Rhee J, Trager LE, Roh JD, Rosenzweig A. Targeting age-related pathways in heart failure. Circ Res. 2020;126:533–51. 32078451 10.1161/CIRCRESAHA.119.315889 PMC7041880

[9] Hastings MH, Zhou Q, Wu C, Shabani P, Huang S, Yu X, et al. Cardiac aging: from hallmarks to therapeutic opportunities. Cardiovasc Res. 2024;00:1–15. 10.1093/cvr/cvae124 PMC12391674 38918884

[10] Xin M, Olson EN, Bassel-Duby R. Mending broken hearts: cardiac development as a basis for adult heart regeneration and repair. Nat Rev Mol Cell Biol. 2013;14:529–41. 23839576 10.1038/nrm3619 PMC3757945

[11] Doll S, Dreßen M, Geyer PE, Itzhak DN, Braun C, Doppler SA, et al. Region and cell-type resolved quantitative proteomic map of the human heart. Nat Commun. 2017;8:1469. 29133944 10.1038/s41467-017-01747-2 PMC5684139

[12] Sen I, Trzaskalski NA, Hsiao YT, Liu PP, Shimizu I, Derumeaux GA. Aging at the crossroads of organ interactions: implications for the heart. Circ Res. 2025;136:1286–305. 40403108 10.1161/CIRCRESAHA.125.325637

[13] López-Otín C, Blasco MA, Partridge L, Serrano M, Kroemer G. Hallmarks of aging: an expanding universe. Cell. 2023;186:243–78. 36599349 10.1016/j.cell.2022.11.001

[14] Fu TE, Zhou Z. Senescent cells as a target for anti-aging interventions: From senolytics to immune therapies. J Transl Int Med. 2025;13:33–47. 40115034 10.1515/jtim-2025-0005 PMC11921816

[15] Zhang F, Wang Y, Zhu J, Wang J, Li Q, Feng J, et al. Region- and cell-type-resolved multiomic atlas of the heart. Mol Cell Proteom. 2025;24:100922. 10.1016/j.mcpro.2025.100922 PMC12139502 39921206

[16] Linscheid N, Santos A, Poulsen PC, Mills RW, Calloe K, Leurs U, et al. Quantitative proteome comparison of human hearts with those of model organisms. PLoS Biol. 2021;19:e3001144. 33872299 10.1371/journal.pbio.3001144 PMC8084454

[17] Chen X, Yu C, Kang R, Tang D. Iron metabolism in ferroptosis. Front Cell Dev Biol. 2020;8:590226. 33117818 10.3389/fcell.2020.590226 PMC7575751

[18] Remesal L, Sucharov-Costa J, Wu Y, Pratt KJB, Bieri G, Philp A, et al. Targeting iron-associated protein Ftl1 in the brain of old mice improves age-related cognitive impairment. Nat Aging. 2025;5:1957–69. 40830655 10.1038/s43587-025-00940-z PMC12532579

[19] Yan S, Lu L, Wu Y, Du Z, Johny E, Dutta P, et al. PCBP2 regulates p16(INK4a)-dependent cellular senescence in response to iron. Aging Cell. 2025;24:e70283. 41216990 10.1111/acel.70283 PMC12686567

[20] de Mezer M, Rogaliński J, Przewoźny S, Chojnicki M, Niepolski L, Sobieska M. et al. SERPINA3: stimulator or inhibitor of pathological changes. Biomedicines. 2023;11:156 36672665 10.3390/biomedicines11010156 PMC9856089

[21] Wang L, Li D, Yao F, Feng S, Tong C, Rao R, et al. Serpina3k lactylation protects from cardiac ischemia reperfusion injury. Nat Commun. 2025;16:1012. 39856050 10.1038/s41467-024-55589-w PMC11760901

[22] Hou M, Huang J, Jia T, Guan Y, Yang F, Zhou H, et al. Deep profiling of the proteome dynamics of pseudomonas aeruginosa reference strain PAO1 under different growth conditions. J Proteome Res. 2023;22:1747–61. 37212837 10.1021/acs.jproteome.2c00785

[23] Yang F, Jia L, Zhou HC, Huang JN, Hou MY, Liu FT, et al. Deep learning enables the discovery of a novel cuproptosis-inducing molecule for the inhibition of hepatocellular carcinoma. Acta Pharm Sin. 2024;45:391–404. 10.1038/s41401-023-01167-7 PMC10789809 37803139

[24] Demichev V, Messner CB, Vernardis SI, Lilley KS, Ralser M. DIA-NN: neural networks and interference correction enable deep proteome coverage in high throughput. Nat Methods. 2020;17:41–44. 31768060 10.1038/s41592-019-0638-x PMC6949130

[25] Perez-Riverol Y, Bandla C, Kundu DJ, Kamatchinathan S, Bai J, Hewapathirana S, et al. The PRIDE database at 20 years: 2025 update. Nucleic Acids Res. 2025;53:D543–d53. 39494541 10.1093/nar/gkae1011 PMC11701690

[26] Feng Z, Fang P, Zheng H, Zhang X. DEP2: an upgraded comprehensive analysis toolkit for quantitative proteomics data. Bioinformatics. 2023;39:btad526 37624922 10.1093/bioinformatics/btad526 PMC10466079

[27] Zhou Y, Zhou B, Pache L, Chang M, Khodabakhshi AH, Tanaseichuk O, et al. Metascape provides a biologist-oriented resource for the analysis of systems-level datasets. Nat Commun. 2019;10:1523. 30944313 10.1038/s41467-019-09234-6 PMC6447622

[28] Langfelder P, Horvath S. WGCNA: an R package for weighted correlation network analysis. BMC Bioinforma. 2008;9:559. 10.1186/1471-2105-9-559 PMC2631488 19114008

[29] Lai P, Liu L, Bancaro N, Troiani M, Calì B, Li Y, et al. Mitochondrial DNA released by senescent tumor cells enhances PMN-MDSC-driven immunosuppression through the cGAS-STING pathway. Immunity. 2025;58:811–25.e7. 40203808 10.1016/j.immuni.2025.03.005

[30] Li N, Huang J, He S, Zheng Q, Ye F, Qin Z, et al. The development of a novel zeolite-based assay for efficient and deep plasma proteomic profiling. J Nanobiotechnol. 2024;22:164. 10.1186/s12951-024-02404-9 PMC11007927 38600601

[31] Ding Y, Zuo Y, Zhang B, Fan Y, Xu G, Cheng Z. et al. Comprehensive human proteome profiles across a 50-year lifespan reveal aging trajectories and signatures. Cell. 2025;188:5763–84. 40713952 10.1016/j.cell.2025.06.047

[32] Huang J, Swieringa F, Solari FA, Provenzale I, Grassi L, De Simone I, et al. Assessment of a complete and classified platelet proteome from genome-wide transcripts of human platelets and megakaryocytes covering platelet functions. Sci Rep. 2021;11:12358. 34117303 10.1038/s41598-021-91661-x PMC8196183

[33] Zhang D, Liu Y, Zhu Y, Zhang Q, Guan H, Liu S, et al. A non-canonical cGAS-STING-PERK pathway facilitates the translational program critical for senescence and organ fibrosis. Nat Cell Biol. 2022;24:766–82. 35501370 10.1038/s41556-022-00894-z

[34] Joshi A, Rienks M, Theofilatos K, Mayr M. Systems biology in cardiovascular disease: a multiomics approach. Nat Rev Cardiol. 2021;18:313–30. 33340009 10.1038/s41569-020-00477-1

[35] Moaddel R, Ubaida-Mohien C, Tanaka T, Lyashkov A, Basisty N, Schilling B, et al. Proteomics in aging research: a roadmap to clinical, translational research. Aging Cell. 2021;20:e13325. 33730416 10.1111/acel.13325 PMC8045948

[36] Dai L, Zhao T, Bisteau X, Sun W, Prabhu N, Lim YT, et al. Modulation of protein-interaction states through the cell cycle. Cell. 2018;173:1481–94.e13. 29706543 10.1016/j.cell.2018.03.065

[37] Salah H, El-Gazzar RM, Abd El-Wahab EW, Charl F. Oxidative stress and adverse cardiovascular effects among professional divers in Egypt. J Occup Environ Hyg. 2023;20:159–69. 36716173 10.1080/15459624.2023.2173364

[38] Keranov S, Dörr O, Jafari L, Liebetrau C, Keller T, Troidl C, et al. SPARCL1 as a biomarker of maladaptive right ventricular remodelling in pulmonary hypertension. Biomarkers. 2020;25:290–95. 32248722 10.1080/1354750X.2020.1745889

[39] Zhang L, Lou Q, Zhang W, Yang W, Li L, Zhao H, et al. CircCAMTA1 facilitates atrial fibrosis by regulating the miR-214-3p/TGFBR1 axis in atrial fibrillation. J Mol Histol. 2023;54:55–65. 36417034 10.1007/s10735-022-10110-9

[40] Consortium AA. Aging Atlas: a multi-omics database for aging biology. Nucleic Acids Res. 2020;49:D825–D30. 10.1093/nar/gkaa894 PMC7779027 33119753

[41] Zhang N, Yu X, Xie J, Xu H. New Insights into the Role of Ferritin in Iron Homeostasis and Neurodegenerative Diseases. Mol Neurobiol. 2021;58:2812–23. 33507490 10.1007/s12035-020-02277-7

[42] Schaum N, Karkanias J, Neff NF, May AP, Quake SR, Wyss-Coray T, et al. Single-cell transcriptomics of 20 mouse organs creates a Tabula Muris. Nature. 2018;562:367–72. 30283141 10.1038/s41586-018-0590-4 PMC6642641

[43] Zarjou A, Black LM, McCullough KR, Hull TD, Esman SK, Boddu R, et al. Ferritin light chain confers protection against sepsis-induced inflammation and organ injury. Front Immunol. 2019;10:131. 30804939 10.3389/fimmu.2019.00131 PMC6371952

[44] Yang X, Ding Y, Sun L, Shi M, Zhang P, Huang Z, et al. Ferritin light chain deficiency-induced ferroptosis is involved in preeclampsia pathophysiology by disturbing uterine spiral artery remodelling. Redox Biol. 2022;58:102555. 36446230 10.1016/j.redox.2022.102555 PMC9706170

[45] Maus M, López-Polo V, Mateo L, Lafarga M, Aguilera M, De Lama E, et al. Iron accumulation drives fibrosis, senescence and the senescence-associated secretory phenotype. Nat Metab. 2023;5:2111–30. 38097808 10.1038/s42255-023-00928-2 PMC10730403

[46] Garza KR, Clarke SL, Ho YH, Bruss MD, Vasanthakumar A, Anderson SA, et al. Differential translational control of 5’ IRE-containing mRNA in response to dietary iron deficiency and acute iron overload. Metallomics. 2020;12:2186–98. 33325950 10.1039/d0mt00192a PMC8057200

[47] Edhager AV, Povlsen JA, Løfgren B, Bøtker HE, Palmfeldt J. Proteomics of the rat myocardium during development of type 2 diabetes mellitus reveals progressive alterations in major metabolic pathways. J Proteome Res. 2018;17:2521–32. 29847139 10.1021/acs.jproteome.8b00276

[48] Victorelli S, Salmonowicz H, Chapman J, Martini H, Vizioli MG, Riley JS, et al. Apoptotic stress causes mtDNA release during senescence and drives the SASP. Nature. 2023;622:627–36. 37821702 10.1038/s41586-023-06621-4 PMC10584674

