# Supplementary material for: Multi-region proteomic mapping identifies FTL1 and SERPINA3K as protective factors in cardiac aging
Source: Cell Death Dis. 2026 May 23;17(1):647. doi: 10.1038/s41419-026-08882-z (PMC13376365; doi:10.1038/s41419-026-08882-z)
Supplement: Supplementary file 1 — supplemental material [file 41419_2026_8882_MOESM1_ESM.docx]

**Supplemental information**

**Multi-region Proteomic Mapping Identifies FTL1 and SERPINA3K as Protective Factors in Cardiac Aging**

**Running title: Cardiac Aging Protection by FTL1 and SERPINA3K**

Jingnan Huang, Xin Sun, Huadong Liu, Kunpeng Li, Xin Liu, Yunmeng Bai, Zhiyu Dong, Xinlei Wu, Xinyi Liu, Lin Jia, Jianlong Yan, Lixin Cheng, Jigang Wang, Lingyun Dai, Qingshan Geng

**Supplemental Methods**

**Proteomics sample preparation**

Cardiac tissues were homogenized in 500 µL of lysis buffer (8 M urea [U5378, Sigma-Aldrich, Darmstadt, Germany], 1% sodium deoxycholate [SDC, 264103, Sigma-Aldrich], 100 mM triethylammonium bicarbonate [TEAB, T7408, Sigma-Aldrich], 1X protease inhibitor cocktail) using a 15-second mechanical disruption step, followed by ice-cold sonication (3 s on, 7 s off) for 1 min. Homogenates were centrifuged at 14,000 × g for 20 minutes at 4°C. The supernatant was collected, and a 10 µL aliquot was reserved for protein quantification via BCA assay (23225, Thermo Fisher Scientific).

Protein aliquots (20 µg) were diluted to a concentration of 0.5 mg/mL using lysis buffer. Samples were reduced with 20 mM dithiothreitol (DTT, D0632, Sigma-Aldrich) at 56°C for 30 minutes and subsequently alkylated with 40 mM iodoacetamide (IAA, I1149, Sigma-Aldrich) in the dark at room temperature for 30 minutes. For protein cleanup, 200 µg of SP3 beads (50 mg/mL stock, GE65152105050250, Sigma-Aldrich) and ethanol (to a final concentration of 50% v/v) were added. Samples were incubated with rotation (1000 rpm) for 5 minutes at room temperature. Following three washes with 200 µL of 80% ethanol, the beads were resuspended in 100 µL of 100 mM TEAB. Protein digestion was performed by adding 0.5 µg each of trypsin and Lys-C, followed by overnight incubation at 37°C. After digestion, the peptide-containing supernatant was collected, concentrated by vacuum centrifugation (SpeedVac, Eppendorf, Hamburg, Germany), and reconstituted in 2% acetonitrile (ACN, 1.59002, Sigma-Aldrich) / 0.1% formic acid (FA, F0654, TCI, Tokyo, Japan). Peptide concentration was determined by absorbance measurement (Nanodrop 2000, Thermo Fisher Scientific). All reagents used here were LC-MS grade and purchased from Sigma-Aldrich (Darmstadt, Germany).

**LC-MS/MS analysis**

Peptides were analyzed by liquid chromatography-tandem mass spectrometry (LC-MS/MS) using an Orbitrap Eclipse Tribrid mass spectrometer (Thermo Fisher Scientific) coupled to an EASY-nLC 1200 system (Thermo Fisher Scientific), as previously described [1, 2]. Samples (500 ng peptides) were loaded onto a 2.5-cm Acclaim™ PepMap™ 100 C18 trap column (5 µm particles, 100 Å pore size; Thermo Fisher Scientific) at 2 µL/min. Separation was achieved on a 75-cm Acclaim™ PepMap™ 100 C18 analytical column (2 µm particles, 100 Å pore size; Thermo Fisher Scientific) at 300 nL/min using the following gradient: 8–28% solvent B (0.1% formic acid in 80% acetonitrile) over 54 min, 28–45% B over 5 min, 45–95% B over 3 min, and 95% B for 12 min. Solvent A was 0.1% formic acid in water. Eluting peptides were ionized by nanoelectrospray ionization (nano-ESI) at 2.3 kV with the ion transfer tube maintained at 320°C. A high-field asymmetric waveform ion mobility spectrometry (FAIMS) device was positioned between the ion source and mass analyzer. FAIMS parameters included inner/outer electrode temperatures of 100°C, carrier gas at default flow, and compensation voltages (CVs) of 0 V and -45 V. MS1: Full scans (350–1650 m/z) were acquired in the Orbitrap at 120,000 resolution (at 200 m/z), with RF lens level at 30%. Automatic gain control (AGC) target was set to 100% and maximum injection time to 100 ms. MS2: Precursor ions (450–750 m/z) were isolated using the quadrupole with 60 non-overlapping 5-m/z windows. Fragmentation occurred via higher-energy collisional dissociation (HCD) at 30% normalized collision energy. MS/MS spectra (145–1450 m/z) were acquired in the Orbitrap at 30,000 resolution, with RF lens at 30%, AGC target of 800%, and maximum injection time of 50 ms.

**Cardiomyocyte-specific delivery of *Serpina3k* (*Sa3k*) gene by AAV9**

The coding sequence of the mouse *Sa3k* gene was cloned into an adeno-associated virus serotype 9 (AAV9) overexpression vector with the cTnT promoter to drive cardiac-specific expression of SA3K fused to a C-terminal 3×Flag tag and a non-fused GFP reporter via a P2A peptide, which was named as pAAV-cTnT-Sa3k-3×Flag-P2A-GFP (Yuyang Biosciences, Shenzhen, China). The pAAV-cTnT-Sa3k-3×Flag-P2A-GFP construct (AAV9-Sa3k) or the corresponding AAV9 empty vector (AAV9-Vec) was administered to mice (n=4 per group) via tail vein injection at a dose of 5 × 10^11^ viral genomes per milliliter (vg/mL) diluted in 100 μL saline. Heart tissues were harvested six weeks post-injection for subsequent analysis. The investigator was blinded to group allocation during the experiment and outcome assessment.

**Construction of knockdown cell lines**

The siRNA oligos for the *Sting* gene (si*Sting*) were purchased from Vigene Biosciences (Jinan, China). Lyophilized si*Sting* is reconstituted in RNase-free buffer (20 μM), then transfected into sh*Sa3k* cells seeded at 30–50% confluency using RFect V2 siRNA transfection reagents (Baidai Bio, Changzhou, China) according to manufacturer specifications. siRNA and transfection reagent are diluted in enhancer buffer, complexed for 15 min, and added to cells at a final concentration of 50 nM. Knockdown efficiency is assessed 48 h post-transfection by Western blot, with scrambled siRNA as a negative control.

**Supplemental** **Figures**

**Figures S1**


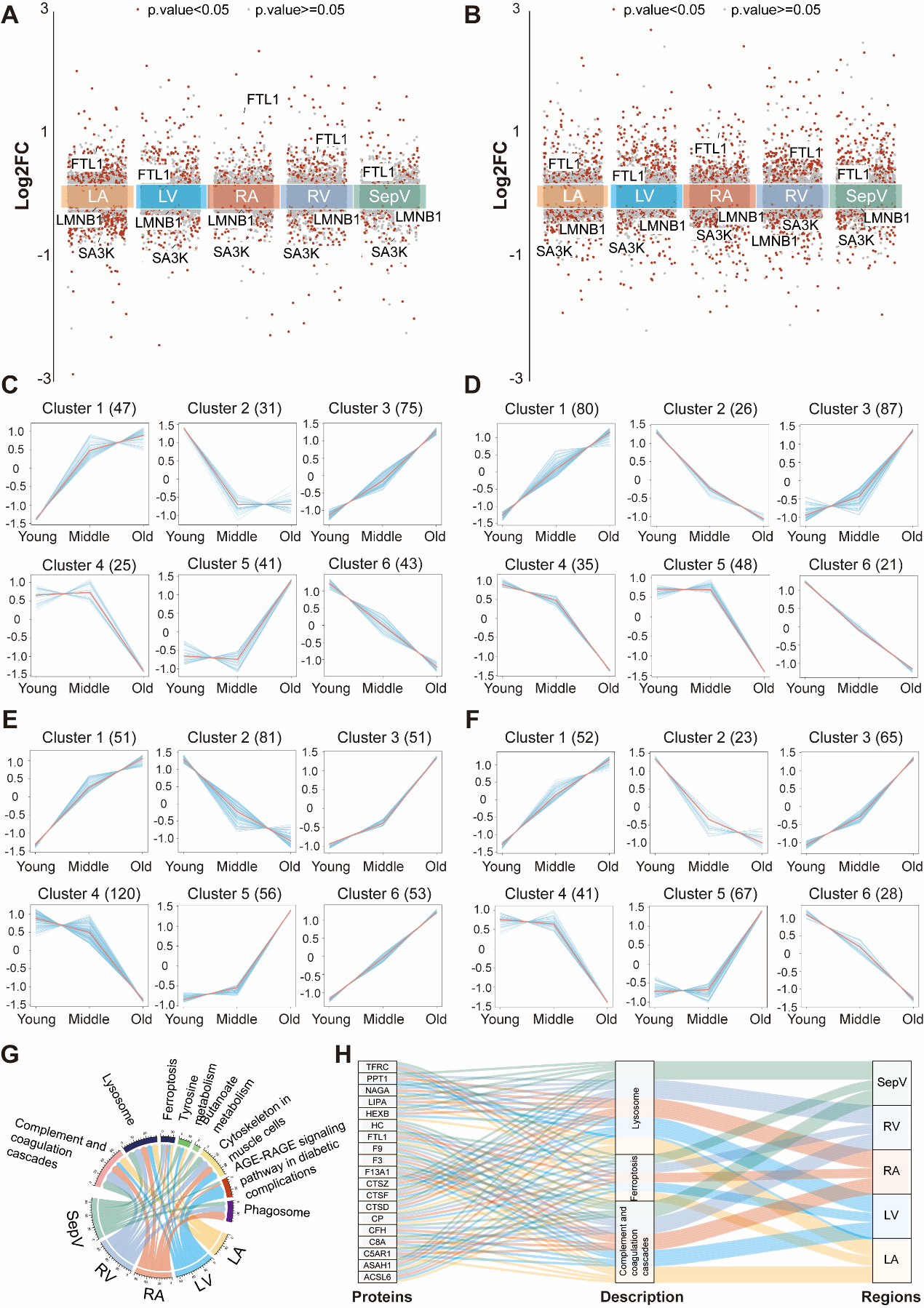


**Figure S1. Aging-associated proteome dynamics across region-resolved mouse heart, related to Figure 3. A** Multi-group volcano plots illustrate the aging-associated proteomic changes by comparing Middle-aged and Young mouse hearts across different cardiac regions, with red dots denoting proteins that exhibit significant dysregulation (|FC| > 1.3, p < 0.05) (n = 5). **B** Multi-group volcano plots illustrate the aging-associated proteomic changes by comparing Old and Middle-aged mouse hearts across different cardiac regions, with red dots denoting proteins that exhibit significant dysregulation (|FC| > 1.3, p < 0.05) (n = 5). **C-F** Mfuzz clustering represents the trajectories of aging-related proteins in the LA **(C)**, RA **(D)**, RV **(E)**, and SepV **(F)**, respectively, where membership is determined by z-score-normalized LFQ intensities (n = 5). **G** Circos plot illustrates the enrichment of region-specific KEGG signaling pathways in the Old vs. Young groups. Correlation arcs connect enriched pathways to various anatomical subregions: LA (cyan), LV (vermilion), RA (azure), RV (olive), SepV (green) (n=5). **H** Sankey diagram illustrating the pathway-associated proteins that are co-enriched in KEGG signaling pathways across the five cardiac regions in aged cohorts (n = 5). Statistical significance was performed using an empirical Bayes moderated t-test. Replicates are cardiac tissues from different mice.

**
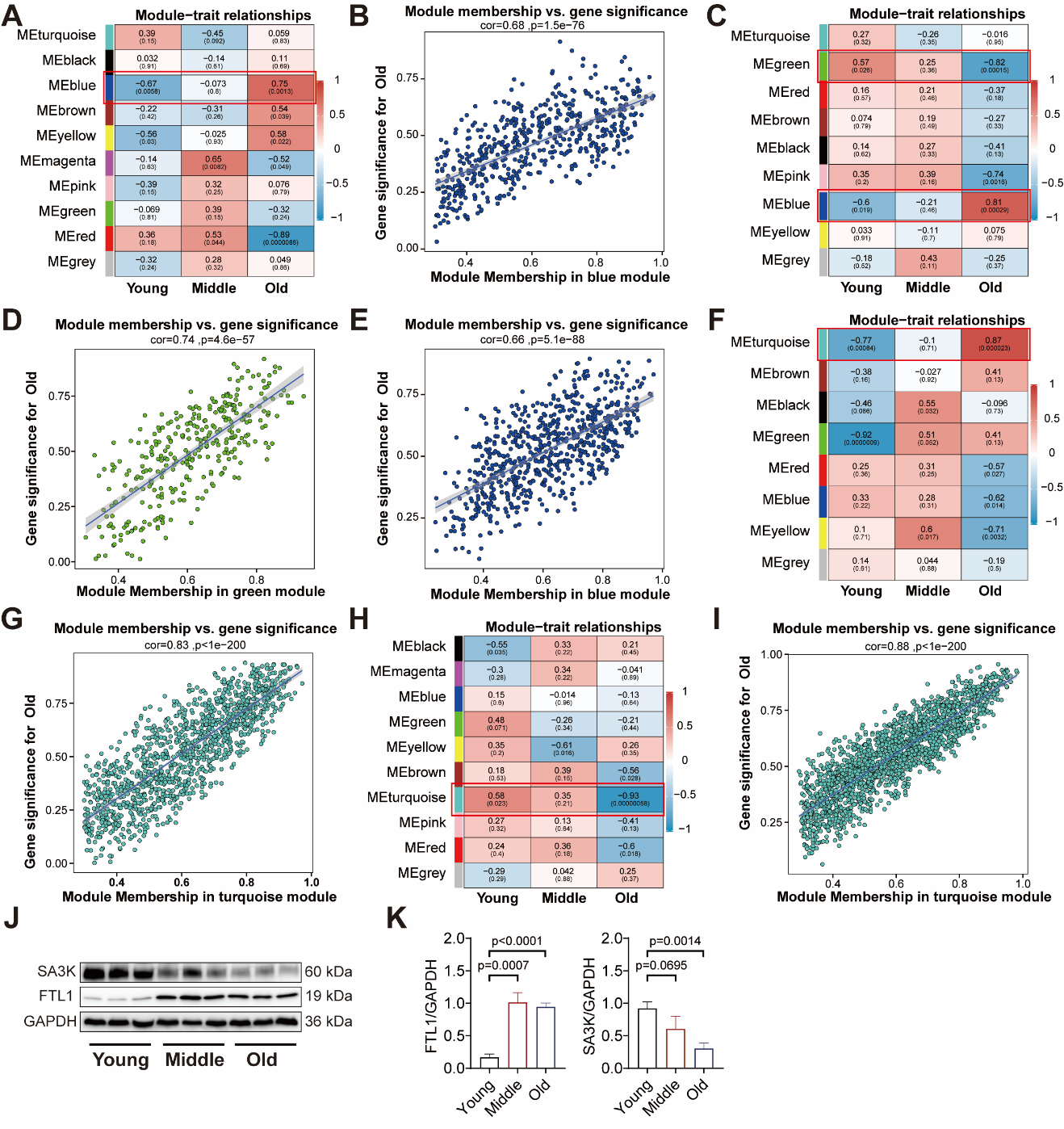
Figure S2**

**Figure S2. FTL1 and SERPINA3K (SA3K) were identified as key regulators of cardiac aging, related to Figure 4. A, C, F, H** Heatmap visualization of the module-trait associations of LA **(A)**, RA **(C)**, RV **(F)**, and SepV **(H)**, respectively. The numbers displayed indicated the correlation coefficients (upper values) and statistical significance (p-values; lower values) between module eigengenes (rows) and cardiac aging (columns). **B, D, E, G, I** The scatter plot depicts the correlation analysis with aging for the module most significantly associated with aging in LA **(B)**, RA **(D and E)**, RV **(G)**, and SepV **(I)**, respectively (n=5).. Replicates are cardiac tissues from different mice. **J** WB analysis shows the protein expression of FTL1 and SA3K in cardiac tissues at different ages. **K** Quantitative analysis quantified the normalized FTL1 and SA3K levels relative to loading controls (GAPDH) (n = 3). Statistical significance was determined using unpaired t-tests.

**
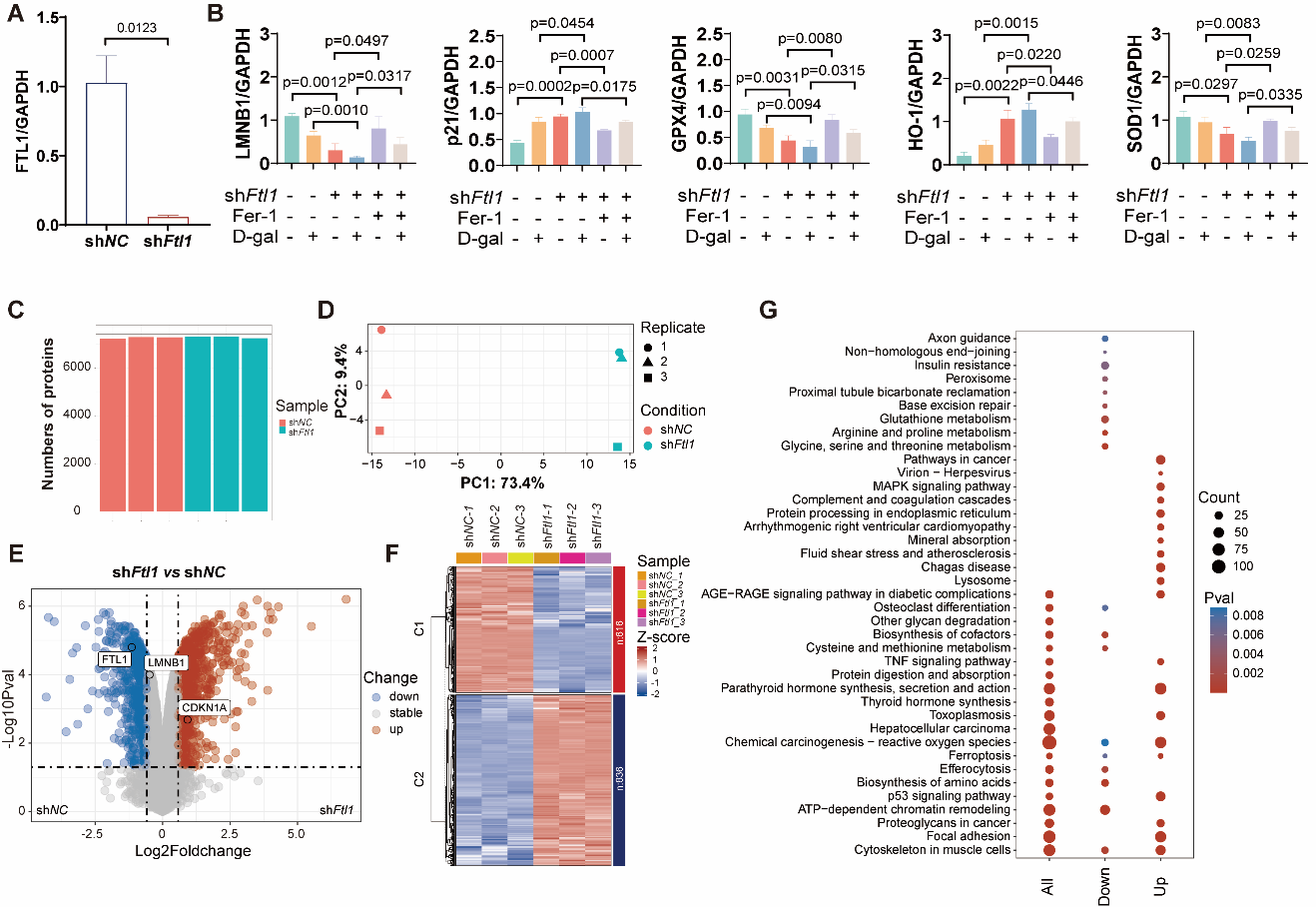
Figure S3**

**Figure S3. Proteomics analysis of sh*Ftl1* cells, related to Figure 5. A** Quantitative analysis quantified the normalized FTL1 levels relative to loading controls (GAPDH) in sh*Ftl1* cells (n = 3). **B** Quantitative analysis quantified the normalized LMNB1, p21, GPX4, HO-1, and SOD1 levels relative to loading controls (GAPDH) in sh*Ftl1* cells (n = 3). **C** Barplot displaying identified protein numbers in sh*Ftl1* cells, and Control cells. **D** Principal component analysis (PCA) showing distinction between sh*Ftl1* cells and sh*NC* cells. **E** Volcano showing DEPs (|FC| > 1.5, p < 0.05) in sh*Ftl1* cells compared to sh*NC* cells (n = 3). **F** Heatmap showing DEPs (|FC| > 1.5, p < 0.05) in sh*Ftl1* cells compared to sh*NC* cells after Z-score scaling. **G** Dot plot linking the DEPs in sh*Ftl1* versus sh*NC* to KEGG signaling pathways. Statistical significance was performed using an empirical Bayes moderated t-test or using unpaired t-tests. Replicates are cells from different cell culture plates.

**Figure S4**

**
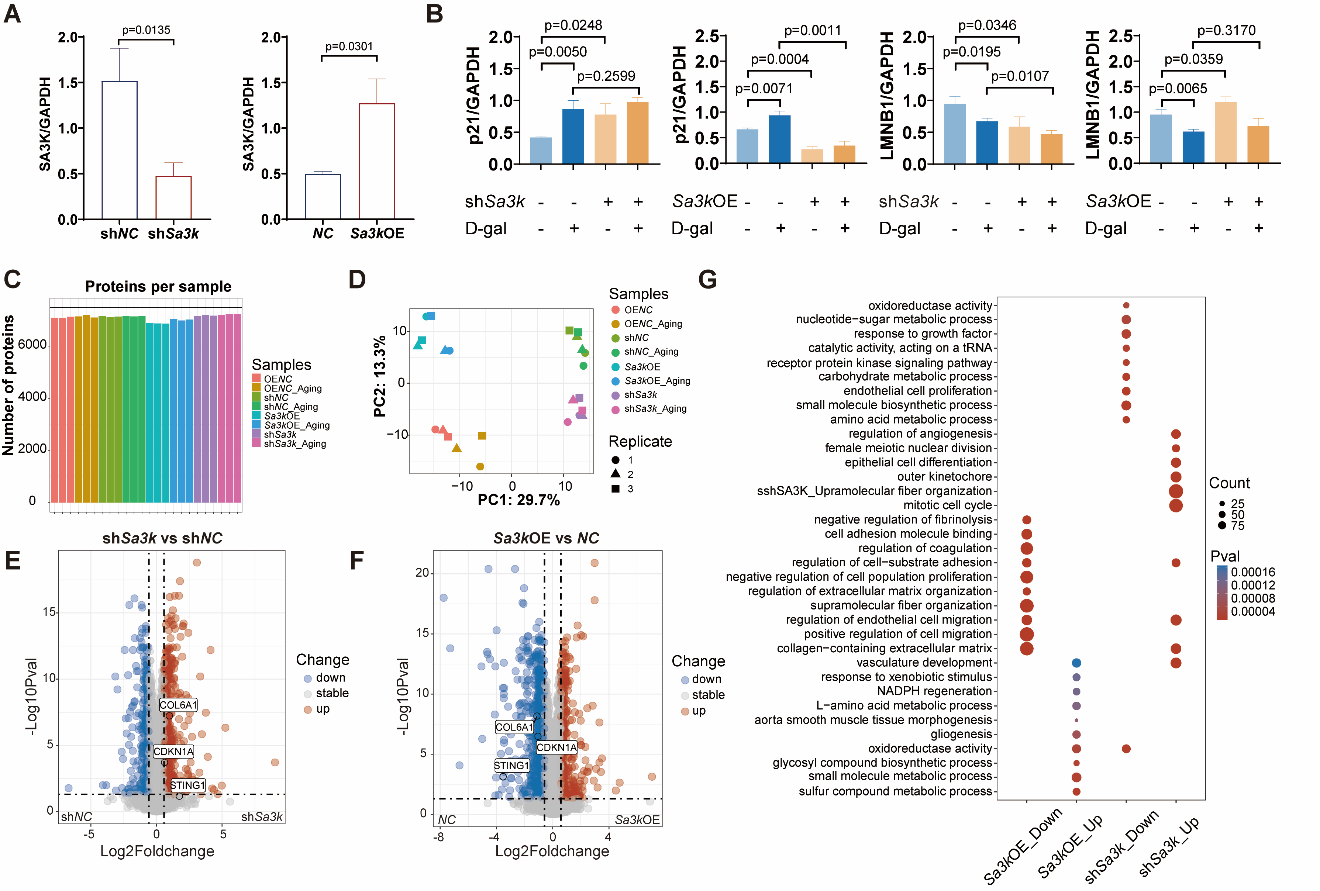
Figure S4. Proteomics analysis of sh*Sa3k* cells and *Sa3k*OE *cells*, related to Figure 6. A** Quantitative analysis quantified the normalized SA3K levels relative to loading controls (GAPDH) in sh*Sa3k* and *Sa3k*OE cells (n = 3). **B** Quantitative analysis quantified the normalized p21 and LMNB1 levels relative to loading controls (GAPDH) in sh*Sa3k* and *Sa3k*OE cells (n = 3). **C** Barplot displaying identified protein numbers in sh*Sa3k*, *Sa3k*OE cells, and Control cells. **D** Principal component analysis (PCA) showing distinction between sh*Sa3k*, *Sa3k*OE cells, and Control cells. **E** Volcano showing DEPs (|FC| > 1.5, p<0.05) in sh*Sa3k* cells compared to sh*NC* cells (n = 3). **F** Volcano showing DEPs (|FC|>1.5, p<0.05) in *Sa3kOE* cells compared to *NC* cells (n = 3). **G** Dotplot linking the DEPs in sh*Sa3k* vs sh*NC*, and *Sa3k*OE vs *NC* to GO biological processes. Statistical significance was performed using an empirical Bayes moderated t-test or using unpaired t-tests. Replicates are cells from different cell culture plates.

**
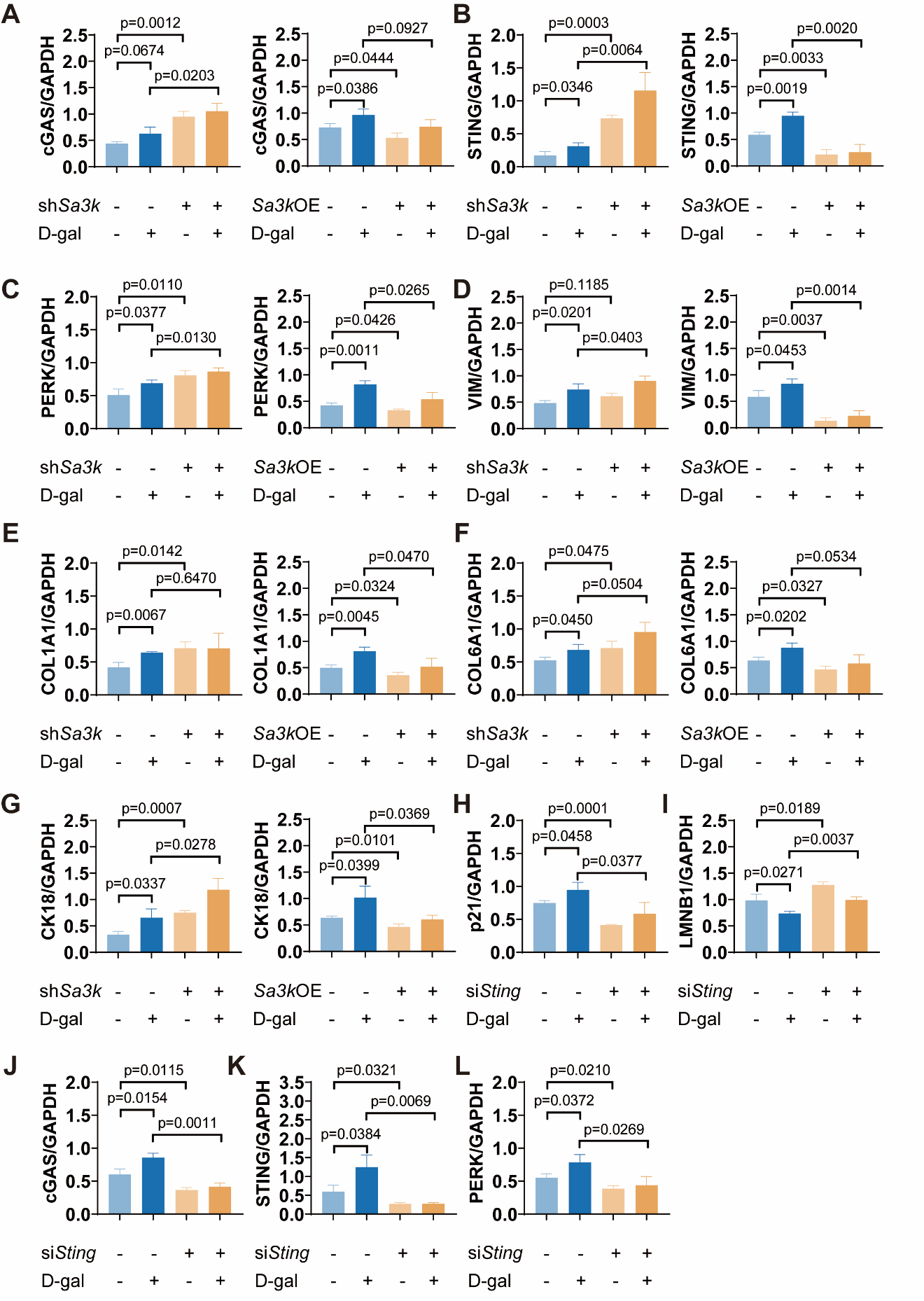
Figure S5**

**Figure S5. Quantitative analysis of WB in sh*Sa3k* cells and *Sa3k*OE *cells*, related to Figure 6. A-G** Quantitative analysis quantified the normalized cGAS **(A)**, STING **(B)**, PERK **(C)**, VIM **(D)**, COL1A1 **(E)**, COL6A1 **(F)**, and CK18 **(G)** levels relative to loading controls (GAPDH) in sh*Sa3k* cells and *Sa3k*OE cells, respectively (n=3). **H-L** Quantitative analysis quantified the normalized p21 **(H)**, LMNB1 **(I)**, cGAS **(J)**, STING **(K)**, and PERK **(L)** levels relative to loading controls (GAPDH) following the si*Sting* treatment in sh*Sa3k* cells (n=3). Statistical significance was determined using unpaired t-tests. Replicates are cells from different cell culture plates.

**Figure S6**

**
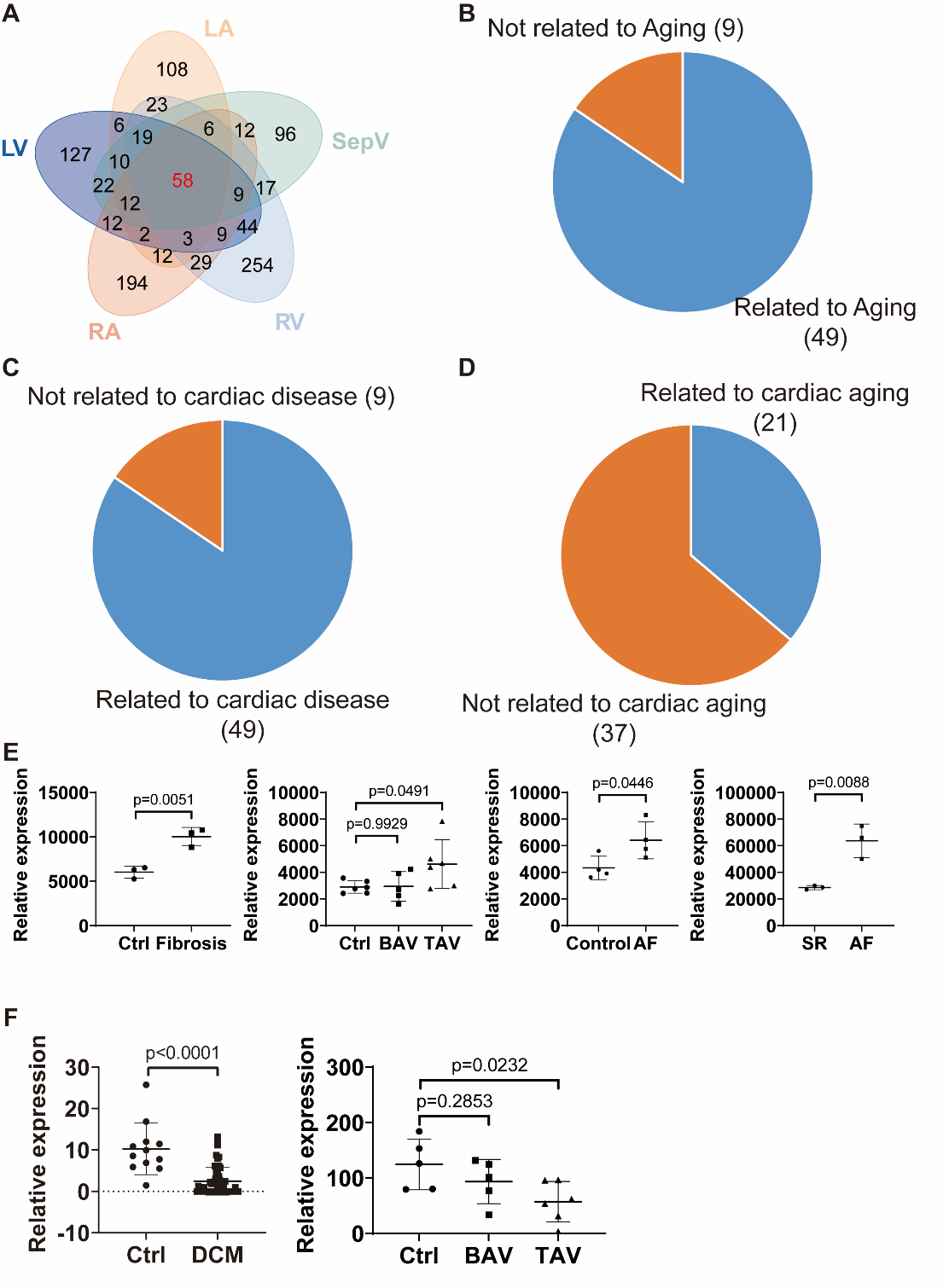
**

**Figure S6. Proteomic profiling identified proteins associated with cardiac aging-related diseases. A** The Venn diagram illustrates 58 overlapping regulated proteins associated with aging across five cardiac regions. **B** Pie chart depicts whether the 58 proteins identified in this study are known aging-related proteins based on the literature. **C** Pie chart depicts whether the 58 proteins identified in this study are known cardiac disease-related proteins based on the literature. **D** Pie chart depicts whether the 58 proteins identified in this study are known cardiac aging-related proteins based on the literature. **E** Relative expression of FTL in human cardiac fibrosis (GSE246298), tricuspid aortic valve (TAV) (GSE148219), and atrial fibrillation (AF) (GSE306533, GSE245886) datasets obtained from GEO. **F** Relative expression of SERPINA3 in human dilated cardiomyopathy (DCM) (GSE245825) and TAV (GSE148219) datasets obtained from GEO. Statistical significance was determined using unpaired t-tests.


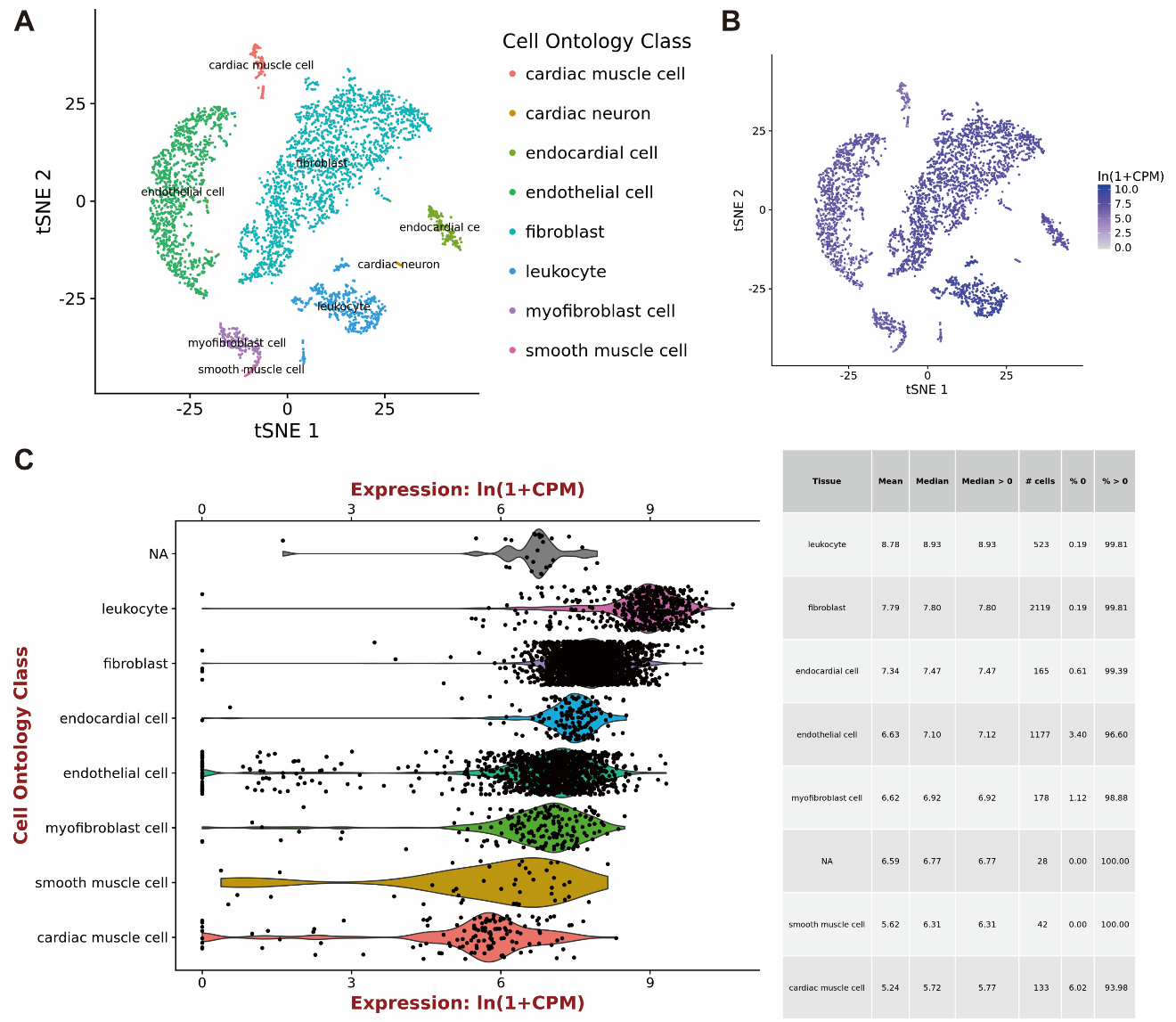
**Figure S7**

**Figure S7. Single-cell transcriptome datasets indicated the expression of *Ftl1* in the mouse heart [3]. A** t-SNE mapping of different cell types in the mouse heart. **B** Transcript expression of *Ftl1* in the mouse heart**. C** The distribution of *Ftl1* expression levels across different cardiac cell types in the mouse hearts.

**
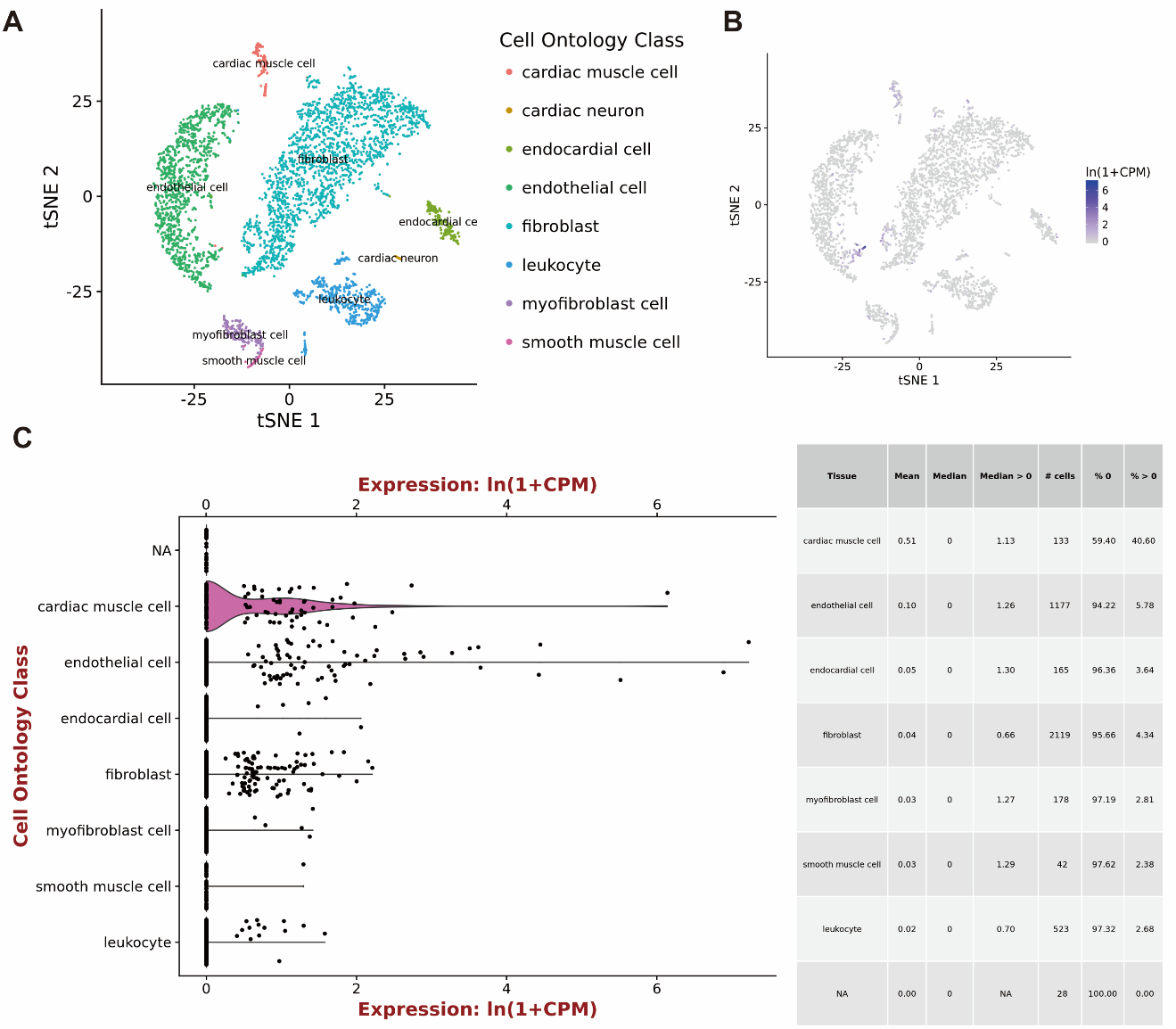
Figure S8**

**Figure S8. Single-cell transcriptome datasets indicated the expression of *Sa3k* in the mouse heart [3]. A** t-SNE mapping of different cell types in the mouse heart. **B** Transcript expression of *Sa3k* in the mouse heart. **C** The distribution of *Sa3k* expression levels across different cell types in the mouse hearts.

**Supplemental tables**

**Table S1. The sequence of shRNAs/siRNA used in this study.**

| Gene name | Forward sequence | Reverse sequence |
| --- | --- | --- |
| Ftl1 | GAAGCCATCTCAAGATGAATG | CATTCATCTTGAGATGGCTTC |
| Serpina3k | GAAATGGACATTGTATTCCAT | ATGGAATACAATGTCCATTTC |
| Sting | CGAAAUAACUGCCGCCUCATT | UGAGGCGGCAGUUAUUUCGTT |

**Table S2. RT-qPCR primers used in this study.**

| Gene name | Forward sequence | Reverse sequence |
| --- | --- | --- |
| Cdkn1a | AACATCTCAGGGCCGAAA | TGCGCTTGGAGTGATAGAAA |
| Lmnb1 | CTGCTGCTCAATTATGCCAAGAAG | GGCAGATAAGGATGCTTCTAGCT |
| Ftl1 | CCATCTGACCAACCTCCGC | CGCTCAAAGAGATACTCGCC |
| Serpina3k | TGAGGAGCTATCGTGCTCTGT | GCCTGTAGTTACTAGCGATGGA |
| Il-1β | TGCCACCTTTTGACAGTGATG | TGATGTGCTGCTGCGAGATT |
| Il-6 | CCGGAGAGGAGACTTCACAG | TCCACGATTTCCCAGAGAAC |
| Cxcl2 | CGGTCAAAAAGTTTGCCTTG | TCCAGGTCAGTTAGCCTTGC |
| β-actin | GTTGTCGACGACGAGCG | GCACAGAGCCTCGCCTT |
| mtDNA Cox1 | GCCCCAGATATAGCATTCCC | GTTCATCCTGTTCCTGCTCC |
| mtDNA Dloop | TCCTCCGTGAAACCAACAA | AGCGAGAAGAGGGGCATT |
| mtDNA Nd1 | CAAACACTTATTACAACCCAAGAACA | TCATATTATGGCTATGGGTCAGG |
| Tert | CTAGCTCATGTGTCAAGACCCTCTT | GCCAGCACGTTTCTCTCGTT |

**Table S3. Antibodies used in this study.**

| Protein name | Vendor | Catalog Number |
| --- | --- | --- |
| CDKN1A/p21 | ABclonal | A2691 |
| LMNB1 | Proteintech | 12987-1-AP |
| FTL1 | Abcam | ab109373 |
| SERPINA3K | Proteintech | 55480-1- AP |
| GPX4 | Proteintech | 67763-1-Ig |
| HO-1/HMOX1 | Proteintech | 10701-1-AP |
| SOD1 | Proteintech | 10269-1-AP |
| cGAS | Proteintech | 29958-1-AP |
| STING | ABclonal | A21051 |
| PERK | Cell Signaling Technology | 3192T |
| VIMENTIN | Cell Signaling Technology | 5741T |
| COL6A1 | Proteintech | 17023-1-AP |
| CK18 | Proteintech | 10830-1-AP |
| COL1A1 | ABclonal | A24112 |
| GAPDH | Proteintech | 60004-1-Ig |
| β-ACTIN | Proteintech | 66009-1-Ig |
| HRP-conjugated Goat Anti-Rabbit IgG(H+L) | Proteintech | SA00001-2 |
| HRP-conjugated Goat Anti-Mouse IgG(H+L) | Proteintech | SA00001-1 |

**Table S4. Correlation among samples from different age stages and different cardiac regions.**

**Table S5. GO enrichment analysis of differentially regulated proteins in different regions.**

**Table S6. Unique proteins identified across different cardiac regions.**

**Table S7. Comprehensive table containing differentially expressed proteins across all age groups, along with GO enrichment analysis, WGCNA analysis, and machine learning analysis of these differentially expressed proteins.**

**Table S8. Correlations with age or cardiac diseases reported in the published literature for proteins that show age-dependent expression across all five cardiac regions.**

**References**

1. Hou M, Huang J, Jia T, Guan Y, Yang F, Zhou H, et al. Deep Profiling of the Proteome Dynamics of Pseudomonas aeruginosa Reference Strain PAO1 under Different Growth Conditions. J Proteome Res. 2023;22:1747-61.

2. Yang F, Jia L, Zhou HC, Huang JN, Hou MY, Liu FT, et al. Deep learning enables the discovery of a novel cuproptosis-inducing molecule for the inhibition of hepatocellular carcinoma. Acta Pharmacol Sin. 2024;45:391-404.

3. Schaum N, Karkanias J, Neff NF, May AP, Quake SR, Wyss-Coray T, et al. Single-cell transcriptomics of 20 mouse organs creates a Tabula Muris. Nature. 2018;562:367-72.
