# Supplementary material for: Multi-region proteomic mapping identifies FTL1 and SERPINA3K as protective factors in cardiac aging
Source: Cell Death Dis. 2026 May 23;17(1):647. doi: 10.1038/s41419-026-08882-z (PMC13376365; doi:10.1038/s41419-026-08882-z)

SDS-PAGE band profile of the PageRular Prestained Protein Ladder (26617, ThermoFisher, USA)

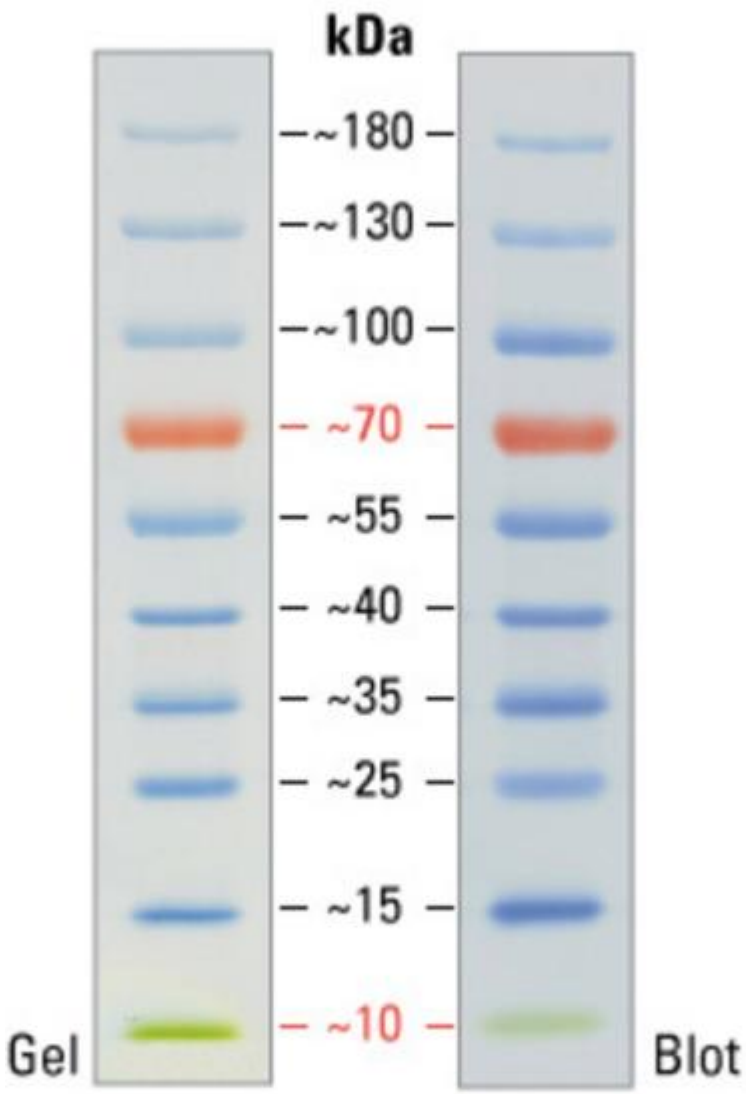

Figure 1E

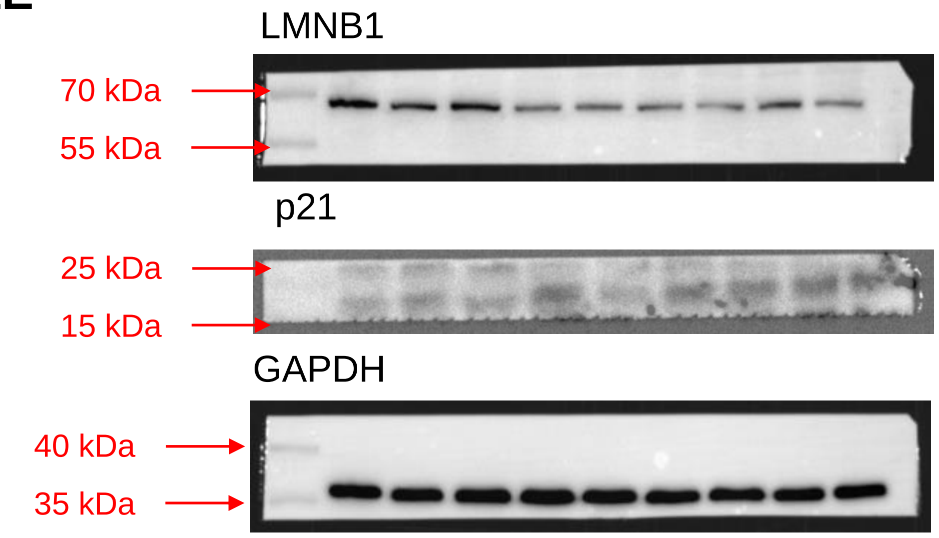

Figure S2J

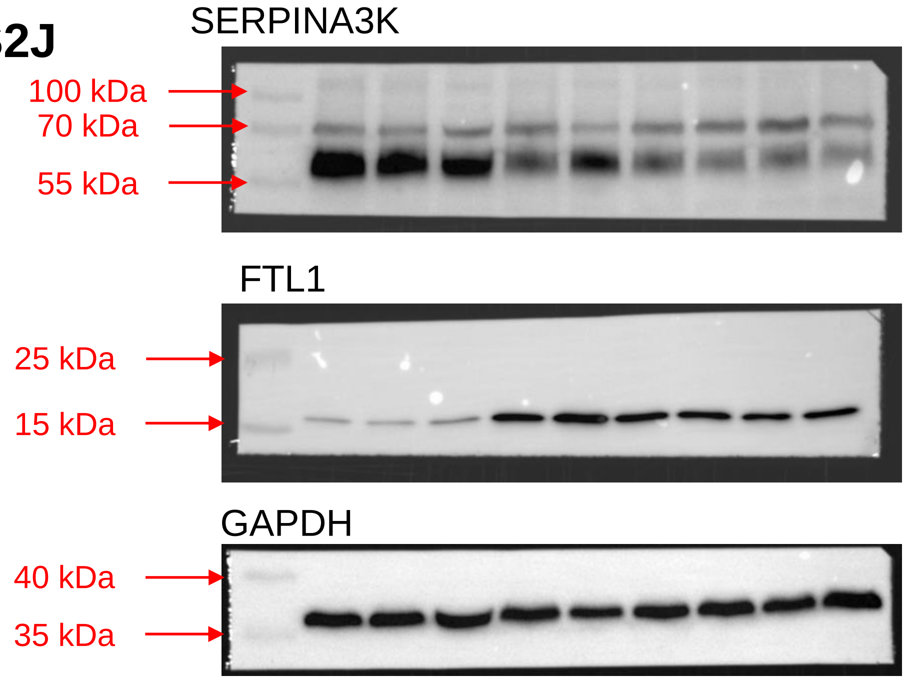

Figure 4H

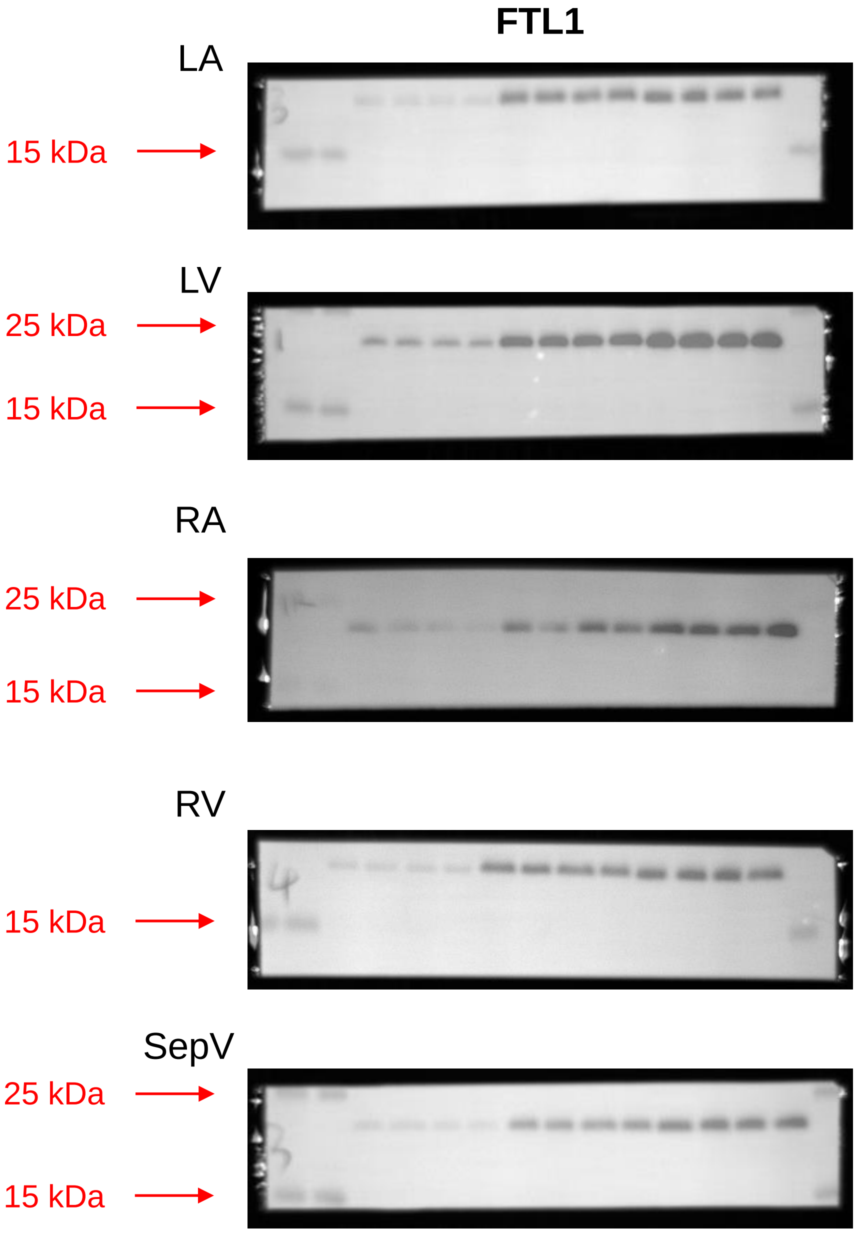

Figure 4I

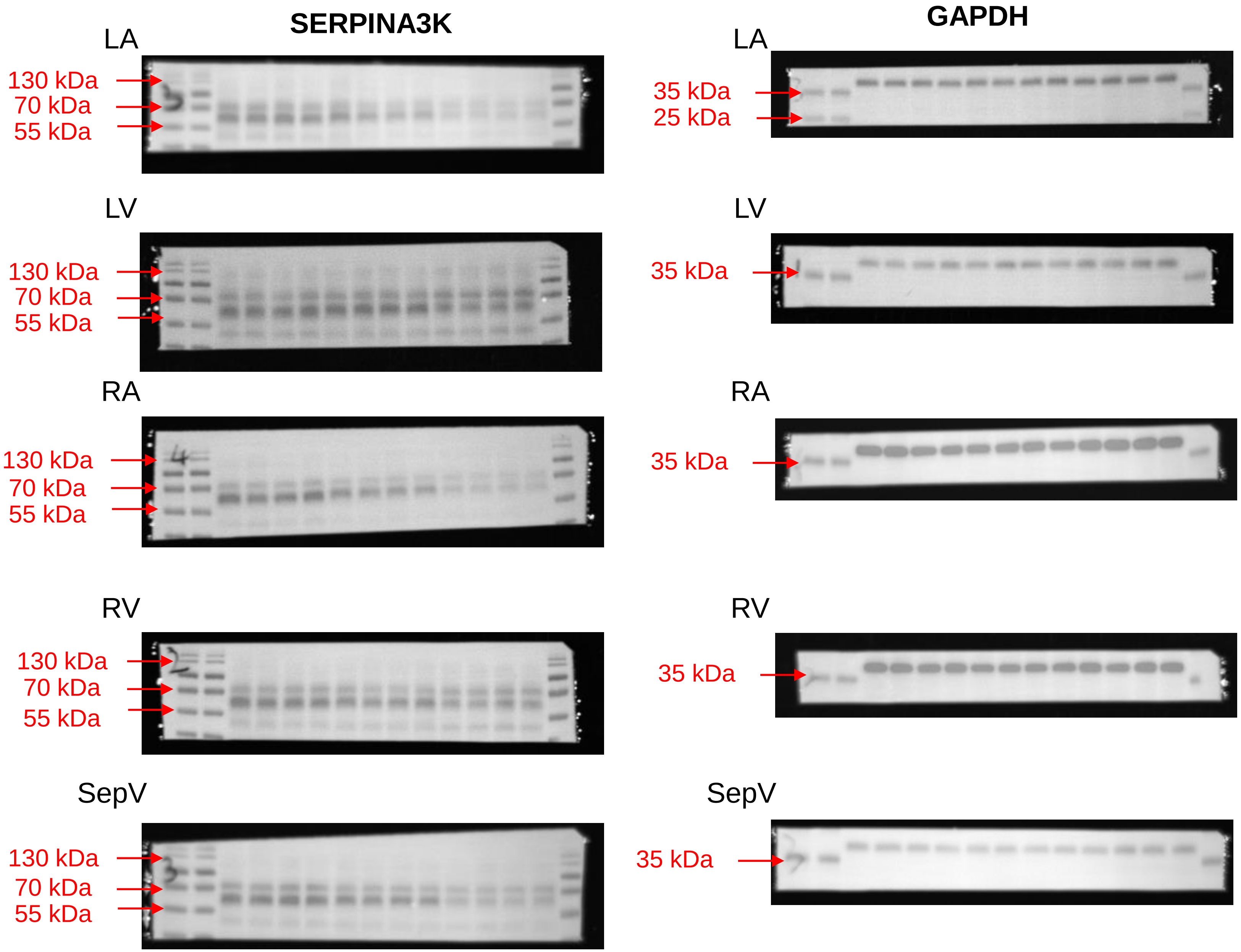

Figure 5A

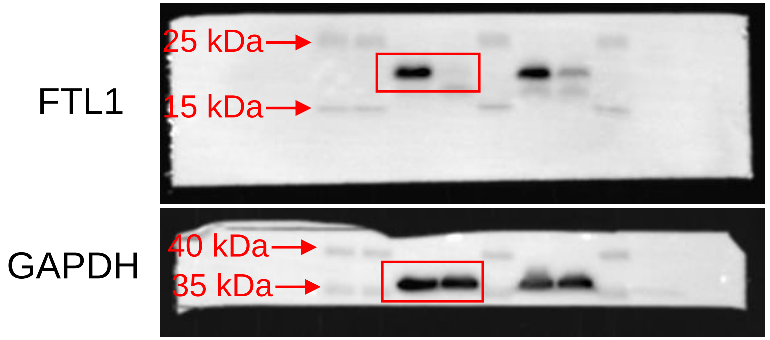

Figure 5G

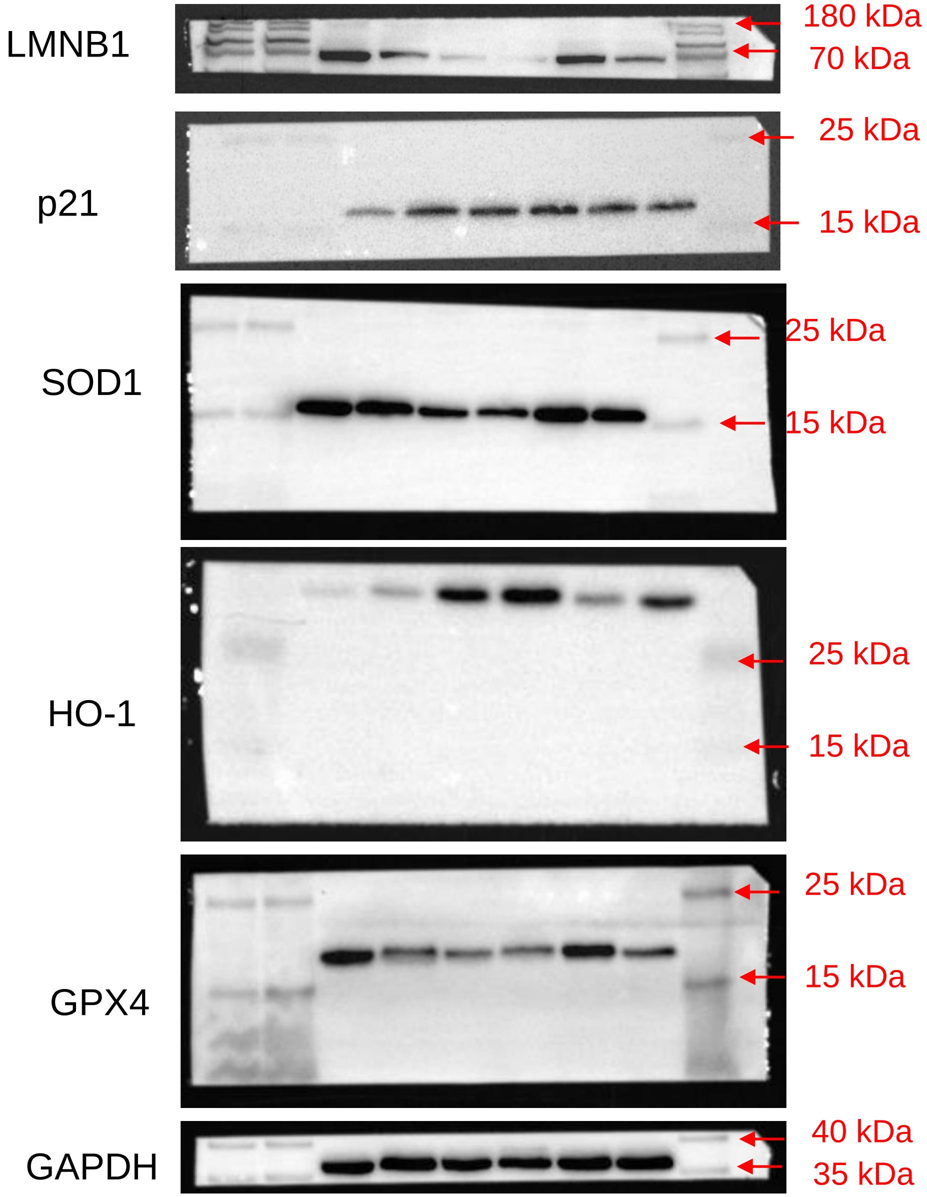

Figure 6A

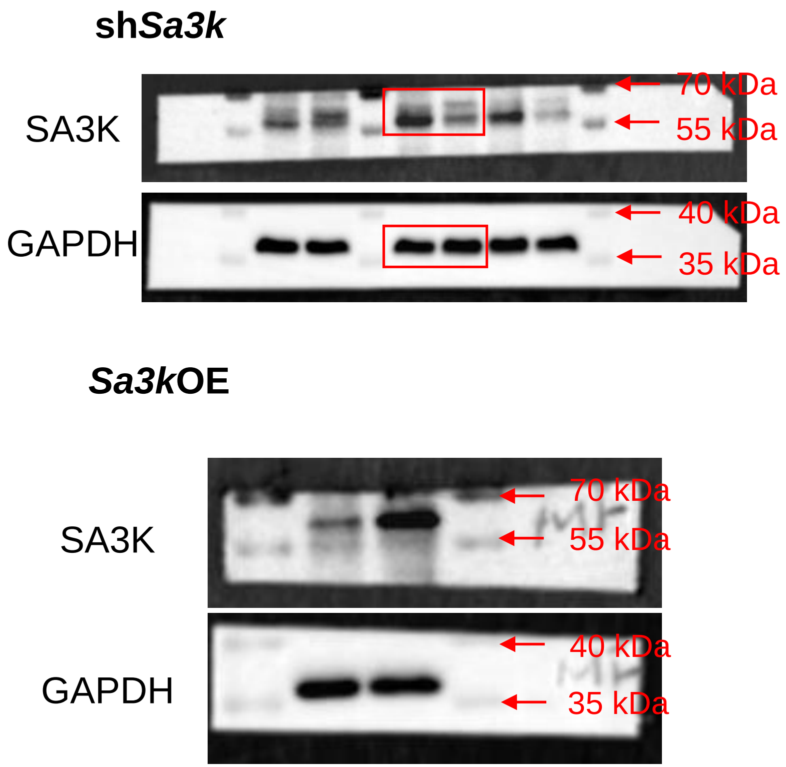

Figure 6E

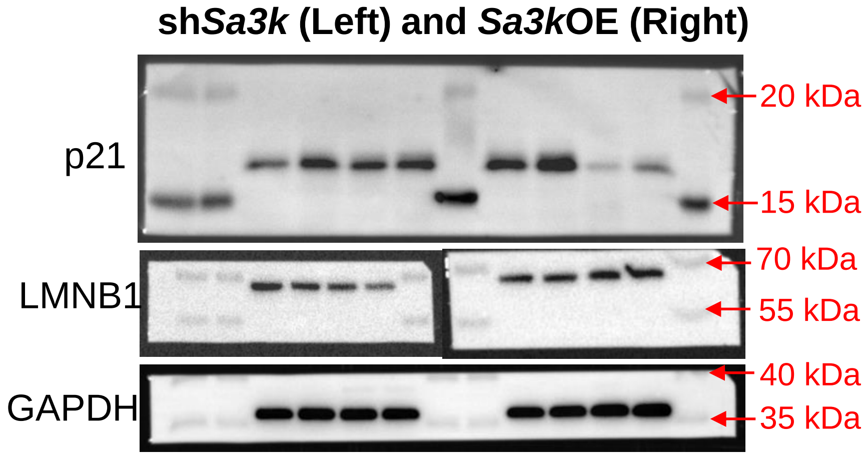

Figure 6H

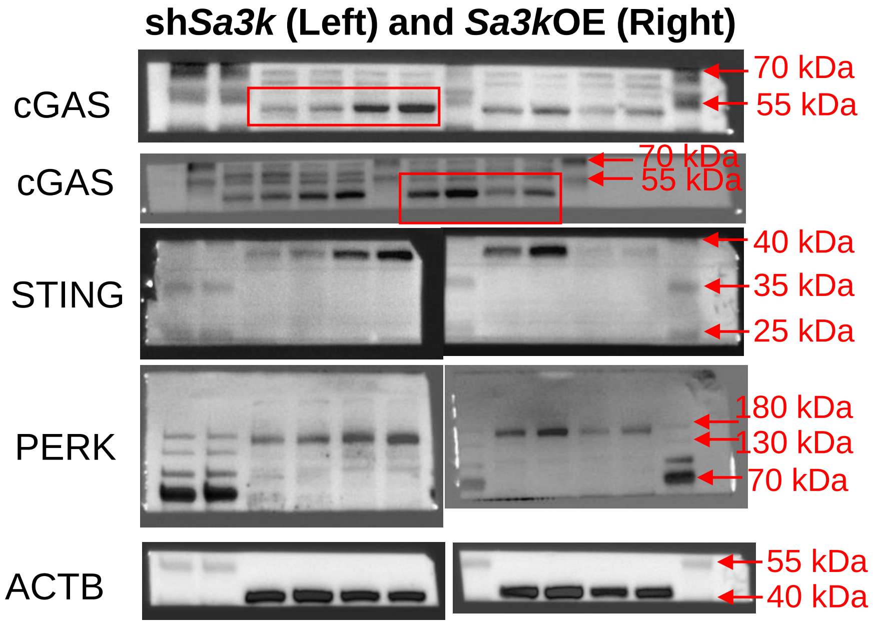

Figure 6I

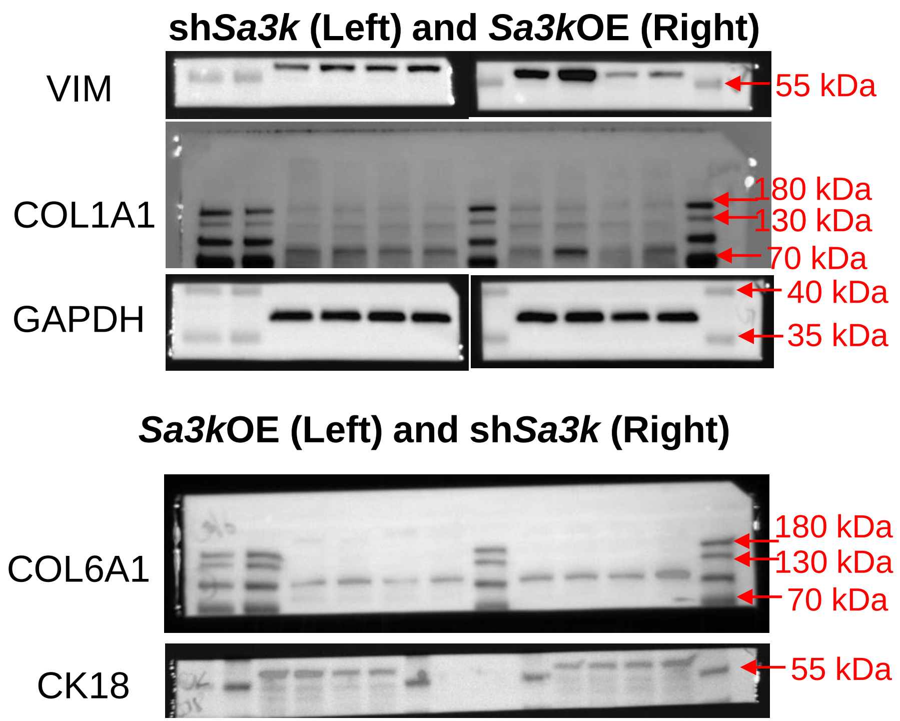

Figure 6N

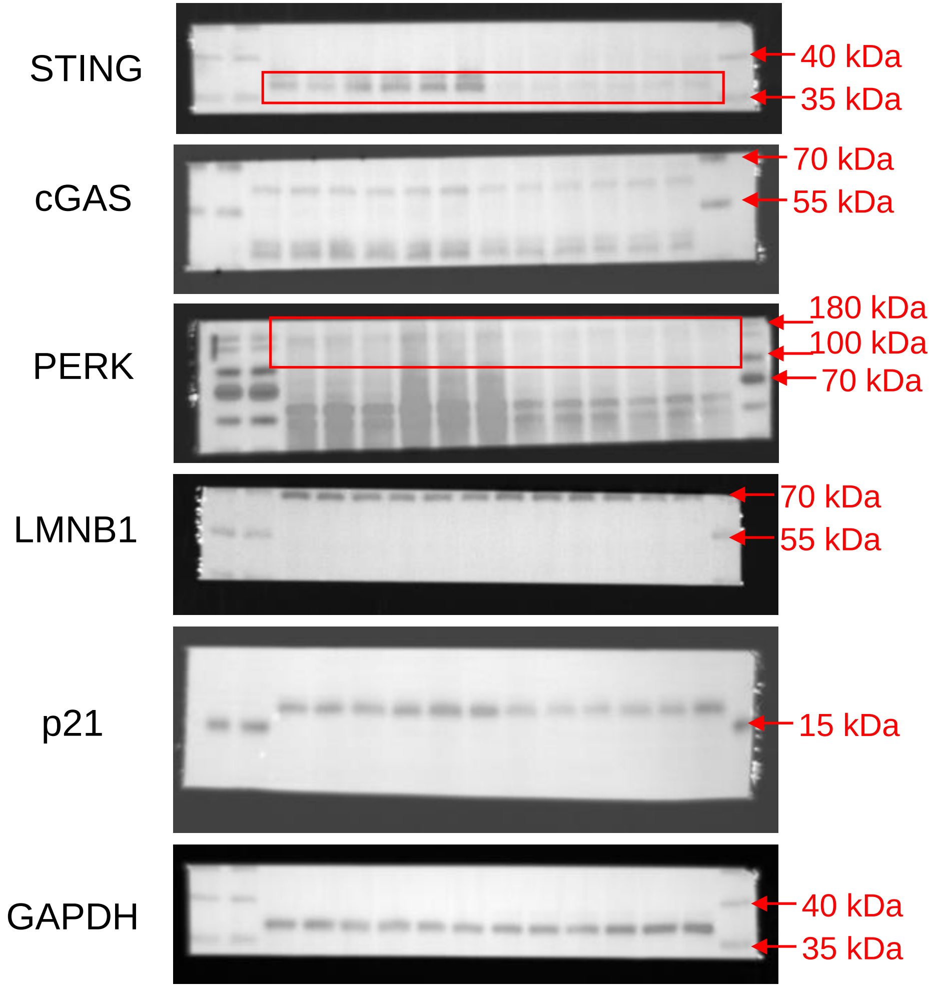

Figure 7B

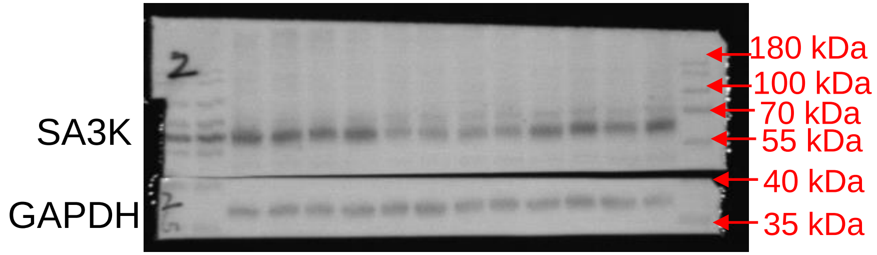

Figure 7E

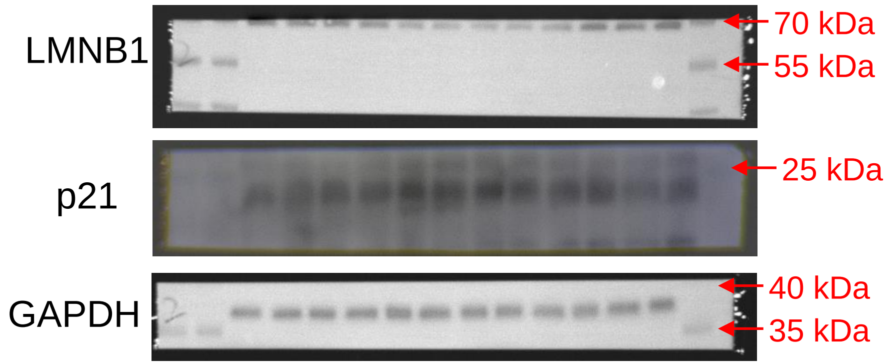

Figure 7G

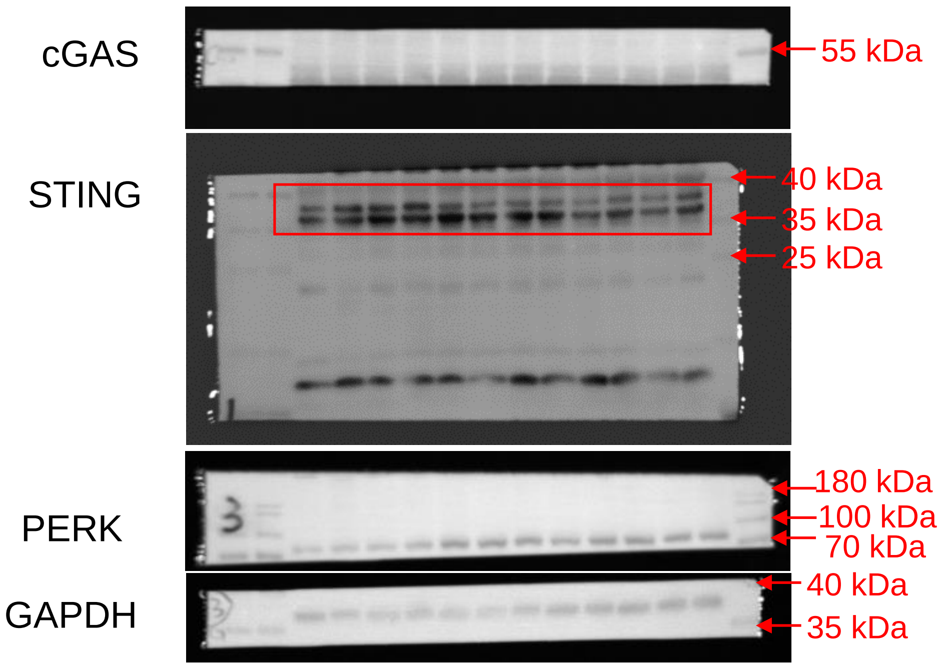

Figure 7I

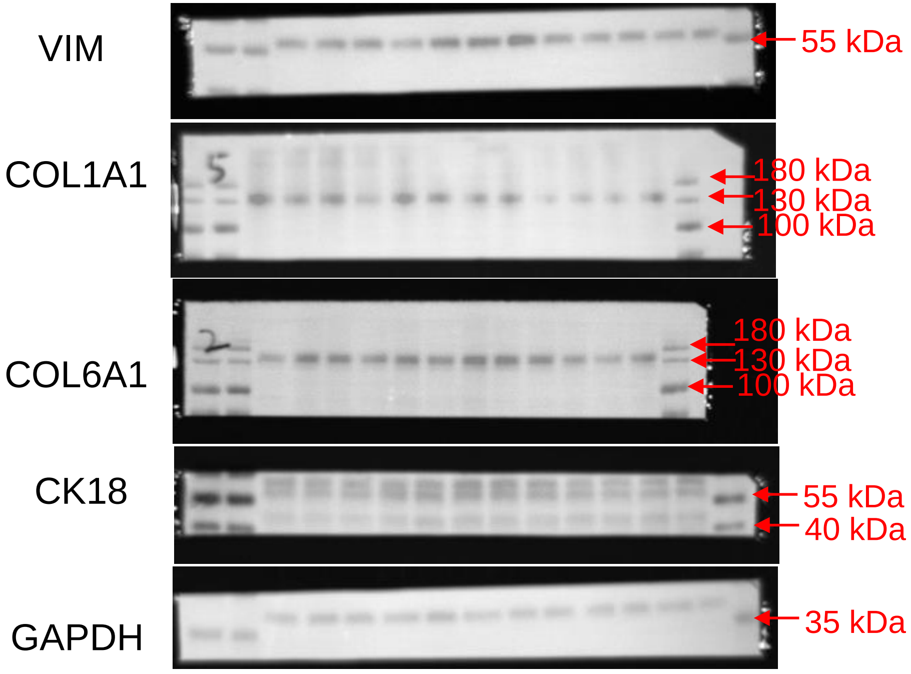

Supplement: Supplementary file 7 — uncropped WB figures [file 41419_2026_8882_MOESM7_ESM.pdf]
